# Uncovering the transcriptional responses of tobacco (*Nicotiana tabacum* L.) roots to *Ralstonia solanacearum* infection: a comparative study of resistant and susceptible cultivars

**DOI:** 10.1186/s12870-023-04633-w

**Published:** 2023-12-06

**Authors:** Hailing Zhang, Muhammad Ikram, Ronghua Li, Yanshi Xia, Weicai Zhao, Qinghua Yuan, Kadambot H. M. Siddique, Peiguo Guo

**Affiliations:** 1 Guangdong Provincial Key Laboratory of Plant Adaptation and Molecular Design, School of Life Sciences, Guangzhou University, Guangzhou, 510006 China; 2 Guangdong Research Institute of Tobacco Science, Shaoguan, 512029 China; 3 Guangdong Provincial Engineering & Technology Research Center for Tobacco Breeding and Comprehensive Utilization, Guangdong Key Laboratory for Crops Genetic Improvement, Crops Research Institute, Guangdong Academy of Agricultural Sciences (GAAS), Guangzhou, 510640 China; 4 The UWA Institute of Agriculture, The University of Western Australia, Perth, WA 6001 Australia

**Keywords:** Tobacco, *Ralstonia solanacearum*, Bacterial wilt, RNA-seq, DEGs, Glutathione, Phenylpropane

## Abstract

**Background:**

Tobacco bacterial wilt (TBW) caused by *Ralstonia solanacearum* is the most serious soil-borne disease of tobacco that significantly reduces crop yield. However, the limited availability of resistance in tobacco hinders breeding efforts for this disease.

**Results:**

In this study, we conducted hydroponic experiments for the root expression profiles of D101 (resistant) and Honghuadajinyuan (susceptible) cultivars in response to BW infection at 0 h, 6 h, 1 d, 3 d, and 7d to explore the defense mechanisms of BW resistance in tobacco. As a result, 20,711 and 16,663 (total: 23,568) differentially expressed genes (DEGs) were identified in the resistant and susceptible cultivars, respectively. In brief, at 6 h, 1 d, 3 d, and 7 d, the resistant cultivar showed upregulation of 1553, 1124, 2583, and 7512 genes, while the susceptible cultivar showed downregulation of 1213, 1295, 813, and 7735 genes. Similarly, across these time points, the resistant cultivar had downregulation of 1034, 749, 1686, and 11,086 genes, whereas the susceptible cultivar had upregulation of 1953, 1790, 2334, and 6380 genes. The resistant cultivar had more up-regulated genes at 3 d and 7 d than the susceptible cultivar, indicating that the resistant cultivar has a more robust defense response against the pathogen. The GO and KEGG enrichment analysis showed that these genes are involved in responses to oxidative stress, plant–pathogen interactions, cell walls, glutathione and phenylalanine metabolism, and plant hormone signal transduction. Among the DEGs, 239 potential candidate genes were detected, including 49 phenylpropane/flavonoids pathway-associated, 45 glutathione metabolic pathway-associated, 47 WRKY, 48 ERFs, eight ARFs, 26 pathogenesis-related genes (PRs), and 14 short-chain dehydrogenase/reductase genes. In addition, two highly expressed novel genes (*MSTRG.61386-R1B-17* and *MSTRG.61568*) encoding nucleotide-binding site leucine-rich repeat (NBS-LRR) proteins were identified in both cultivars at 7 d.

**Conclusions:**

This study revealed significant enrichment of DEGs in GO and KEGG terms linked to glutathione, flavonoids, and phenylpropane pathways, indicating the potential role of glutathione and flavonoids in early BW resistance in tobacco roots. These findings offer fundamental insight for further exploration of the genetic architecture and molecular mechanisms of BW resistance in tobacco and solanaceous plants at the molecular level.

**Supplementary Information:**

The online version contains supplementary material available at 10.1186/s12870-023-04633-w.

## Background

*Nicotiana tabacum* (2*n* = 48) is an economically major non-edible cash crop, with its dried leaves used for industrial products, including cigarettes, cigars, and shisha tobacco, and stems used for biofuel production [1–4]. However, bacterial wilt (BW), a soil-borne disease caused by *R. solanacearum* that seriously damages leaf quality and causes yield losses in tobacco, is distributed in all tobacco production areas, especially warm-temperate or tropical and subtropical areas [5–7]. The disease also attacks other Solanaceae crops such as potato, eggplant, tomato, pepper, and sweet potato [6–10]. Production losses due to BW varies from 10–20% in peanut [6], 10–15% in potato [11], 20–50% in chili [12, 13], and 20–30% in ginger [14]. Tobacco bacterial wilt (TBW) disease incidence significantly reduces tobacco production by 10–30% worldwide [7, 15–17], reaching 15–35% [6] in the major growing areas of Sichuan, Guangdong, Hunan, Guizhou, and Hubei provinces in China [18]. However, TBW control is a global problem, with traditional methods (chemical control, tobacco-rice rotation, and soil fumigation) inadequate for reducing yield and economic losses [16, 19]. Therefore, studies are needed to investigate BW's defense mechanism or genetic basis and identify genes responsive to the infection to develop BW-resistant tobacco cultivars.

During evolution, Solanaceae plants developed complex defense mechanisms against pathogens [20], such as inhibitory substances, metabolites (alkaloids, phenols, etc.), and hormones [21]. Several quantitative trait loci (QTLs) have been reported for BW resistance in tobacco [7, 16, 22]; for example, Lan et al. [16] identified eight QTLs,*qBWR17a/17b, qBWR2, qBWR6, qBWR12,* and *qBWR24a/24b/24c*. In another study, using bi-parental mapping, four QTLs (qBWR-3a/3b and qBWR-5a/5b) were detected for TBW resistance [23]. Habe et al. [24] used QTL mapping analysis in potato, detecting five QTLs (*qBWR1, qBWR2, qBWR3, qBWR4*, and *qBWR5*) in response to BW infection. Similarly, Nguyen et al. Chae et al. [12, 25], and Zai et al. [26] reported 8, 31, and 14 significant SNPs for BW resistance in tomato, pepper, and common beans, respectively, using association mapping. These QTLs/SNPs, especially those with significant effects [27], will assist marker-assisted breeding of BW-resistant cultivars [7, 28]. Thus, Few QTLs/QTNs associated with BW resistance in tobacco have been identified compared to other Solanaceae species: pepper, tomato, potato, and eggplant [6, 11–14].

Moreover, transcriptomics, metabolomics, genomics, and proteomics are practical and diverse methodologies for elucidating and comprehensively understanding complex biological mechanisms under various stressful conditions in plants [17, 29–31]. Of these, transcriptomics analysis with RNA-seq or microarrays has been used to unravel the molecular basis and genes associated with the specific biological processes in solanaceous crops, which could provide BW resistance for developing superior cultivars [9, 30]. In light of previous studies, transcriptomics and proteomics analysis showed that the methionine cycle (MTC), phenylpropanoid biosynthesis, glutathione metabolism, and gamma-aminobutyric acid biosynthesis pathways played essential roles in tomato BW [9, 29, 32]. [32] used resistant and susceptible tomato genotypes to demonstrate that 140 up-regulated DEGs were related to hormones, lignin, and pathogenesis in the resistant genotype, while no changes occurred in the susceptible genotype. Similarly, Li et al. [8] identified 302 DEGs associated with potato BW, of which 81 were considered for BW resistance involved in signal transduction, terpenoids, pathogen recognition, hypersensitive response, and protection [8].

For TBW, Gao et al. [9] identified 158 and 835 DEGs in resistant and susceptible accessions, respectively, in a seedling root transcriptome study, while Shi et al. [29] reported that indole-3-acetic acid (IAA) and abscisic acid (ABA) plays significant roles in BW resistance in transcriptomics and metabolomics studies. A study reported the up-regulation of 6,233 genes in tobacco-resistant cultivars in response to BW infection. These genes were enriched in cell walls, ABC transporters, endocytosis, and glutathione metabolism [33]. Similarly, Li et al. [17] and Pan et al. [33] used the tobacco stem transcriptome to detect genes, identifying the phenylpropanoid metabolic pathway for tobacco defense against BW. Furthermore, many studies have reported the root transcriptome in pepper [34], potato [35], eggplant [36], and tomato [37] as compared to tobacco. For example, root transcriptomes at 1, 3, and 5 d identified 115 genes in response to pepper BW infection [34]. Moreover, transcription factors (TFs) [38], including WRKY, ERFs, NAC, MYB, bHLH, and bZip, and gene families like P450, MAPK, and DRGs identified in Solanaceae crops are involved in pathogen resistance [17, 29, 39]. Further, recent studies have reported candidate genes associated with plant defense against BW, including *StMKK1* in potato [40], *CaNAC2c* in pepper [41], *SlNAP1* in tomato [42], and *NbPDKs* in tobacco [43]. Limited transcriptomic studies have been conducted to investigate the genes involved in tobacco's resistance to *R. solanacearum*. Therefore, exploring and analyzing defense-related genes in tobacco through transcriptomics can provide valuable insights into the interaction between tobacco and *R. solanacearum*.

Controlling tobacco bacterial wilt in China is difficult due to its high severity and limited availability of resistant tobacco cultivars. Recently, we identified 52 candidate genes and 38 quantitative trait nucleotides (QTNs) using association mapping of 94 tobacco accessions with 126,602 SNPs [7]. Findings from the studies mentioned above in other solanaceous species can serve as a reference for advancing tobacco breeding efforts. However, the genetic basis and molecular mechanism of BW resistance in tobacco may differ from those in other solanaceous species. Until now, few transcriptome profiling studies of BW resistance have been reported in tobacco. Therefore, in this study, we used RNA-seq to analyze and compare transcriptomes in the roots of D101 [resistant (R)] and Honghuadajinyuan [susceptible (S)] cultivars at early (6 h, 1 d) and late (3 d and 7 d) stage of seedlings after inoculation. The study aimed to understand the early and late response of gene expression to *R. solanacearum* infection in roots and to identify the genes conferring root resistance to *R. solanacearum* through gene ontology (GO), Kyoto Encyclopedia of Genes and Genomes (KEGG), and functional enrichment analysis. We identified significant divergence between the resistant and susceptible cultivars in their pathogen responses and also identified potential genes associated with BW resistance in tobacco plants for gene cloning, molecular studies, and breeding purposes.

## Results

### Phenotypic divergence of tobacco cultivars under *R. solanacearum* infection

**Fig. 1:**
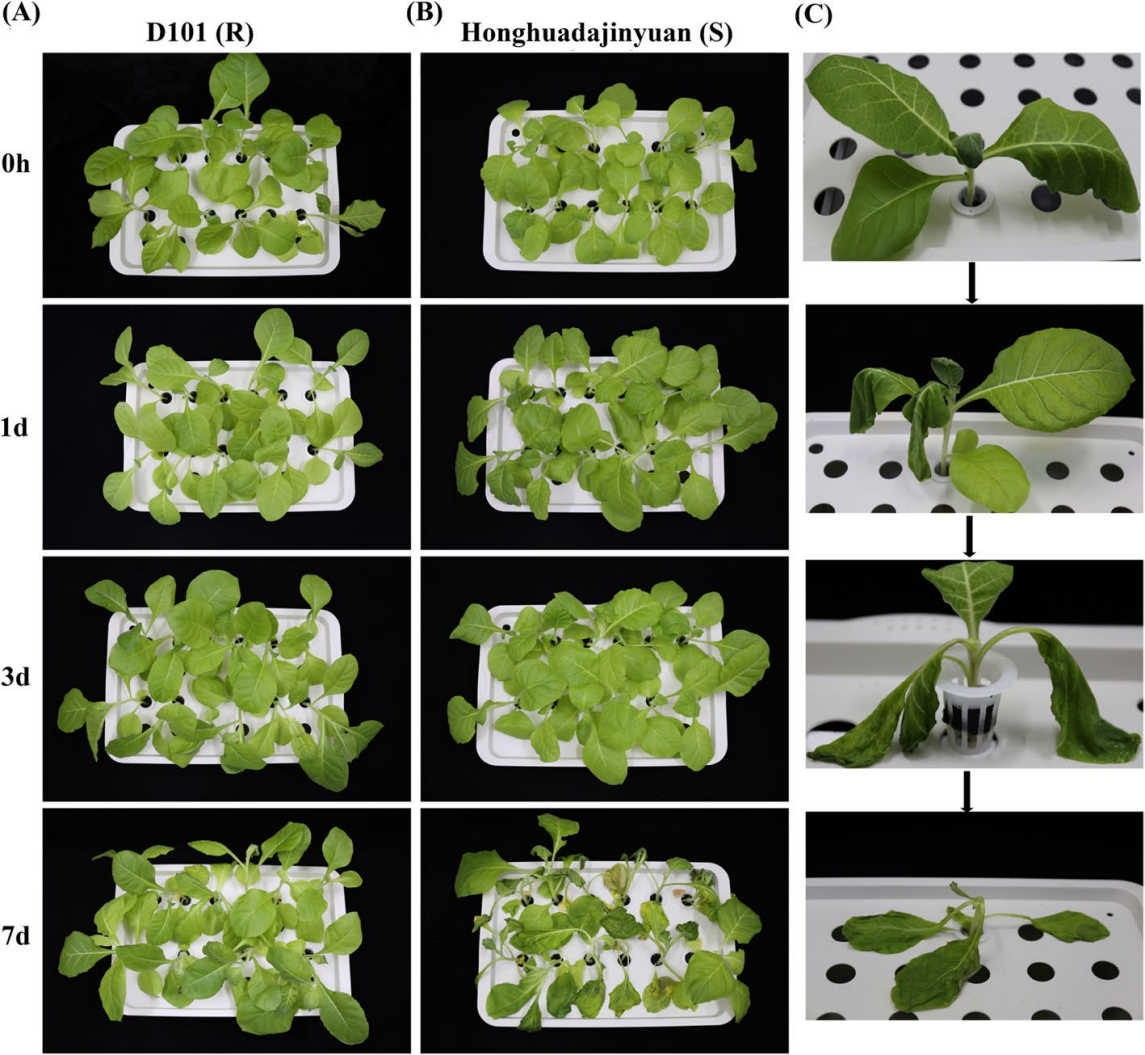
Disease symptoms in D101 (resistant; R) and Honghuadajinyuan (susceptible; S) tobacco cultivars after inoculation with *R. solanacearum*. Seedlings of R (**A**) and S (**B**) cultivars at 0 d, 1 d, 3 d, and 7 d after infection. The leaves show clear disease symptoms at 3 and 7 days in both cultivars as compared to 1 and 3 days, but the leaves of the S cultivar at 7 d show black color and plant death. **C** Bacterial wilt symptoms in leaves and stem of tobacco seedlings

Firstly, we performed disease assays to determine the response of tobacco cultivars (D101 and Honghuadajinyuan) to bacterial wilt. We observed a significant difference between accessions in disease symptoms at 3 d and 7 d. Notably, the TBW symptoms (leaf wilting) started to appear in Honghuadajinyua after 3 d exposure to infection, with no symptoms in D101 (Fig. 1A). At 7 d, Honghuadajinyua stems looked black, with some plant death (Fig. 1B-C), whereas D101 seedlings had no similar or obvious symptoms (Fig. 1A). The roots of R and S cultivars showed clear disease symptoms at 7 d compared to control condition (Additional file 2: Fig. S1). These findings suggest that the D101 cultivar is highly resistant and Honghuadajinyua is susceptible to TBW infection (Fig. 1B).

### Overview of RNA sequencing

**Fig. 2:**
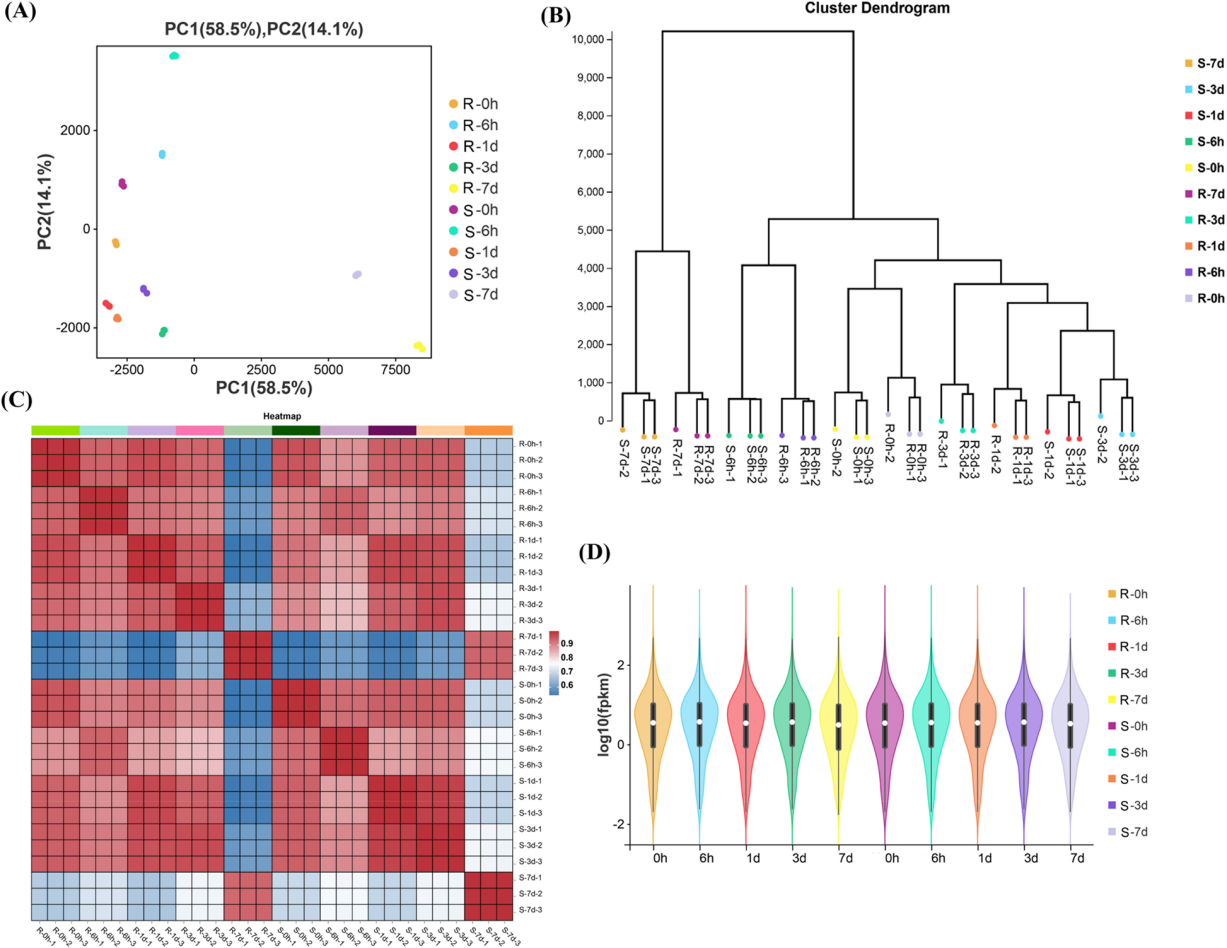
Overview of tobacco root transcriptomic data based on bacterial wilt resistant (R) and susceptible (S) tobacco cultivars at five time points. **A** Principal component analysis represents 81.70% overall variation in expression; **B** Cluster analysis using FPKM values of each sample with replicates indicates the similarities between the biological replicates of both cultivars; **C** Correlation analysis between resistant and susceptible tobacco cultivars at four time points between the samples; **D** log_10_ transformation of FPKM values at four time-points show the differences between the samples at each time points

A total of 1,458.74 million raw reads were collected with an average of 48.62; each library had 40.81–58.89 million raw reads (Additional file 1: Table S1). A total of 1,453.41 (99.64%) million clean reads ranging from 40.66 (99.58%) to 58.67 (99.68%) were obtained after removing adopters, low-quality reads, and unknown bases (Additional file 1: Table S1). The clean reads data was high quality, with a Q30 base rate ranging from 92.78–94.94%, with an average GC content of 42.90%. Further, clean reads were aligned with the reference genome of tobacco (Nitab 4.5), with ~ 1,314.89 (90.80%) million total reads mapped to the genome (Additional file 1: Table S1), of which 1,235.21 (85.29%) million reads were uniquely mapped to transcripts, with an average 41.17 (85.29%) and range 33.62–52.03 (68.44–90.39%), indicating that the sequenced data were suitable for further analysis. Finally, 81,534 genes were identified, of which 69,500 were known, and 12,034 were new. The PC analysis showed 81.70% overall variation, of which PC1 and PC2 had 67.60% and 14.10%, respectively, variation in expression (Fig. 2A). The hierarchical clustering analysis showed similarities between the biological replicates of each R and S cultivar sample (Fig. 2B). Through PC and clustering analysis, the resistant and susceptible accessions clustered from each other, with their replicates located nearby (Fig. 2A-B). The R-7d and S-7d after infection were clustered separately from other time points. All the time points of R and S cultivars except R-7d and S-7d had significant positive correlations (*r* = 0.60–0.70) with other time points (Fig. 2C). The boxplots showed differences between different time points (Fig. 2D).

### Mining differentially expressed genes (DEGs) under bacterial wilt

**Fig. 3:**
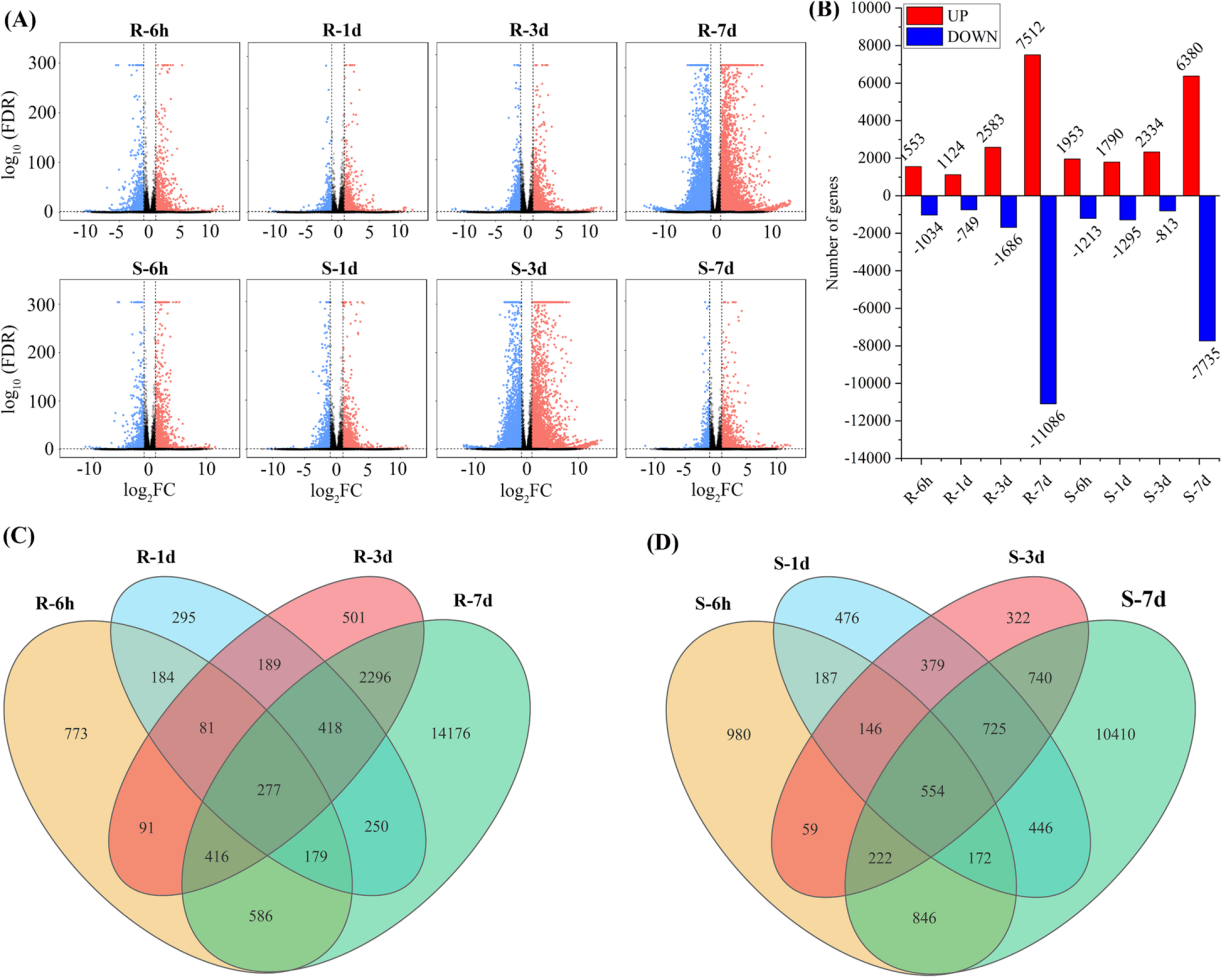
Analysis of differentially expressed genes in resistant (R) and susceptible (S) tobacco cultivars in response to bacterial wilt infection. **A** Volcano plots represent significant genes in R and S cultivars at 6 h, 1 d, 3 d, and 7 d relative to CK-0 h, with log_2_FC values transformed into –log_10_ (FDR). Blue and red dots indicate up-regulated and down-regulated DEGs (log2FC ≥  ± 1, FDR ≤ 0.05); black dots represent non-significant. **B** The number of genes differentially expressed at different time points. The highest number of DEGs were identified at 7 d in R and S cultivars. **C**-**D** The Venn diagram shows the number of unique and common DEGs in R and S tobacco cultivars at each time point

A pairwise comparison of gene expression levels between inoculation and control treatments identified 23,568 (19,130 known and 4,438 novels) DEGs in R and S at four time points (Fig. 3A and Additional file 1: Table S2). In the R cultivar, 20,711 (87.88%) genes were differentially expressed, of which 2,587 (1,553 up and 1,034 down), 1,873 (1,124 up and 749 down), 4,269 (2,583 up and 1,686 down), and 18,598 (7,512 up and 11,086 down) DEGs were identified at R-6 h, R-1d, R-3d, and R-7d, respectively, and 277 DEGs were common at all stages (Fig. 3B-C and Additional file 1: Table S2). Likewise, S samples at S-6 h, S-1d, S-3d, and S-7d had 3,166 (1,953 up and 1,213 down), 3,085 (1,790 up and 1,295 down), 3,147 (2,334 up and 813 down), and 14,115 (6,380 up and 7,735 down) DEGs, respectively (Fig. 3B), with 554 commonly identified at all time-points (Fig. 3D and Additional file 1: Table S2). In addition, 157 DEGs were commonly induced at 6 h, 1 d, 3 d, and 7 d in R and S cultivars (Fig. 3B-C). The R cultivar had 1.10 and 1.17 times more up-regulated genes than the S cultivar at 3 d and 7 d, respectively, while the S cultivar had 1.26 and 1.59 times more up-regulated genes than the R cultivar at 6 h and 1 d (Additional file 1: Table S2). These results indicate that TBW infection had a more substantial regulatory effect on gene regulation at 3 d and 7 d in R cultivars and 6 h and 1 d in S cultivars.

### Gene ontology (GO) enrichment analysis of DEGs

**Fig. 4:**
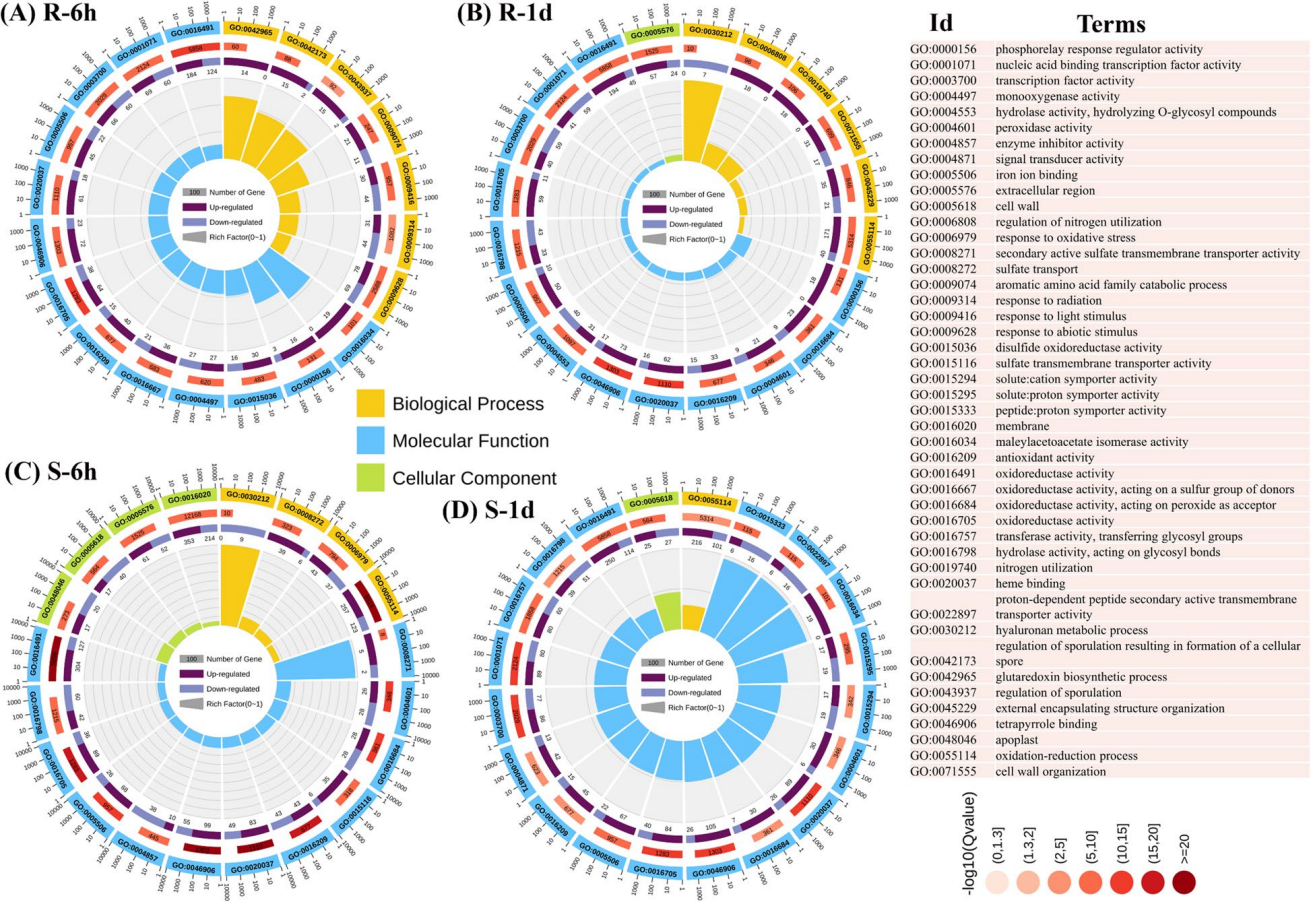
Gene ontology (GO) enrichment analysis of up- and down-regulated genes in bacterial wilt resistant (R) and susceptible (S) tobacco cultivars at 6 h and 1 d. The top significant GO terms of the three categories are shown at R-6 h (**A**), R-1d (**B**), S-6 h (**C**), and S-1d (**D**) at *P* ≤ 0.05. The circular diagram represents the enrichment, GO terms with the number of up- and down-regulated genes. The outer circular line indicates the GO ID of each term, the middle circular line indicates the q-value of enrichment with the total number of genes, and the inner circular lines indicate the up-regulated (purple) and down-regulated (blue) number of genes

The GO enrichment analysis revealed that 1,236 and 1,121 GO terms were significantly enriched in R and S cultivars, respectively. Information on the DEGs and their corresponding GO terms is in Fig. 4, Additional file 2: Fig. S2–S5 and Additional file 1: Table S3. For the R cultivar, 1,554, 1,206, 2,712, and 12,024 DEGs at R-6 h, R-1d, R-3d, and R-7d, respectively, were involved in 235, 288, 452, and 746 GO terms (Fig. 4A-B, Additional file 2: Fig. S2, S3 and Additional file 1: Table S3), including ‘GO:0005975 carbohydrate metabolic,’ ‘GO:0071554 cell wall organization or biogenesis,’ ‘GO:0016491 oxidoreductase activity,’ ‘GO:0006979 response to oxidative stress,’ ‘GO:0005618 cell wall,’ ‘GO:0006749 glutathione metabolic process,’ ‘GO:0009698 phenylpropanoid metabolic process,’ and ‘GO:0046271 phenylpropanoid catabolic process’ (Fig. 4 and Additional file 1: Table S3). For the S cultivar, 2,012, 1,926, 2,014, and 9,096 DEGs were significantly enriched for 263, 530, 532, and 576 GO terms at S-6 h, S-1d, S-3d, and S-7d, respectively (Fig. 4, Additional file 2: Fig. S3–S5 and Additional file 1: Table S3), including ‘GO:0016740 transferase activity,’ ‘GO:0008017 microtubule binding,’ ‘GO:0048046 apoplast,’ ‘GO:0015631 tubulin binding,’ ‘GO:0005874 microtubule,’ ‘GO:0046527 glucosyltransferase,’ ‘GO:0003700 transcription factor,’ ‘GO:0004871 signal transducer activity,’ ‘GO:0006749 glutathione metabolic process,’ and ‘GO:0009812 flavonoid metabolic process,’ (Additional file 2: Fig. S3–S5 and Additional file 1: Table S3). Additionally, genes involved in the defense-related process, such as pathogen-associated molecular pattern perception, signaling, and the activation of defense-related enzymes and proteins, were enriched in the R cultivar (Fig. 4A-B and Additional file 1: Table S3). Interestingly, the S cultivar had fewer genes enriched in the above-mentioned biological processes, suggesting that these processes are less active or less efficient in the susceptible cultivar (Fig. 4C-D and Additional file 1: Table S3), which could help explain why this cultivar is more susceptible to TBW infection than the R-resistant cultivar. Finally, the top 10 potential up-regulated and downregulated DEGs, their expression pattern, and GO terms at all time points were identified, which might play an essential role in disease resistance (Table 1).

**Table 1:** List of up-regulated and down-regulated DEGs, GO, and their expression spectrum under bacterial wilt infection

Gene ID	Gene name	R-6 h	R-1d	R-3d	R-7d	S-6 h	S-1d	S-3d	S-7d	GO terms/Functional annotation
		**Log2FC**	**Log2FC**	**Log2FC**	**Log2FC**	**Log2FC**	**Log2FC**	**Log2FC**	**Log2FC**	
Nitab4.5_0003084g0040	NPF5.7	2.07	2.37	2.92	2.64	3.09	3.46	3.71	4.20	GO:0016020//membrane; GO:0015333//peptide activity; GO:0022857//transmembrane transporter activity
Nitab4.5_0005779g0060	CKX5	2.16	3.43	3.42	2.54	3.40	4.22	4.27	3.53	GO:0005576//extracellular region; GO:0000166//nucleotide binding; GO:0003824//catalytic activity; GO:0005488//GO:0010817//regulation of hormone levels; GO:0071704//organic substance metabolic process
Nitab4.5_0002400g0010	CYP94C1	2.21	2.54	2.04	2.00	2.46	3.11	2.61	2.63	GO:0005506//iron ion binding; GO:0016705//oxidoreductase activity; GO:0020037//heme binding; GO:0055114//oxidation–reduction process
Nitab4.5_0000152g0170	RFS1	2.23	2.55	2.23	5.02	2.19	2.21	2.19	3.74	GO:0016758//transferase activity; GO:0005975//carbohydrate metabolic process; GO:0044238//primary metabolic process
MSTRG.75244	GLP5A	2.90	2.30	3.32	2.85	3.66	2.99	3.67	5.14	GO:0016623//oxidoreductase activity; GO:0046914//transition metal ion binding; GO:0044710//single-organism metabolic process
Nitab4.5_0008666g0010	TIC32B	3.27	5.13	5.64	4.12	2.98	6.40	7.00	5.69	GO:0004757//sepiapterin reductase activity; GO:0050662//coenzyme binding; GO:0051287//NAD binding; GO:0015995//chlorophyll biosynthetic process; GO:0055114//oxidation–reduction process
Nitab4.5_0002337g0010	ATJ11	3.40	3.11	3.40	4.83	2.81	2.97	2.57	3.27	GO:0005515//protein binding; GO:0006457//protein folding; GO:0009408//response to heat
Nitab4.5_0000713g0070	KUA1	4.40	2.47	3.21	8.17	4.50	2.66	3.31	7.64	GO:0005622//intracellular; GO:0005623//cell; GO:0044444//cytoplasmic part; GO:0044464//cell part; GO:0009058//biosynthetic process; GO:0009059//macromolecule biosynthetic process
Nitab4.5_0003171g0010	PPO	4.58	3.30	3.44	5.78	6.00	5.00	7.01	9.26	GO:0004097//catechol oxidase activity; GO:0016491//oxidoreductase activity; GO:0055114//oxidation–reduction process
Nitab4.5_0000402g0160	10HGO	4.60	4.20	4.40	8.91	3.11	3.26	4.22	5.24	GO:0005737//cytoplasm; GO:0016491//oxidoreductase activity
Nitab4.5_0006169g0020	ZIP3	-2.68	-1.80	-3.26	-3.68	-1.94	-1.38	-1.60	-2.16	GO:0016020//membrane; GO:0046873//metal ion transmembrane transporter activity; GO:0030001//metal ion transport; GO:0055085//transmembrane transport
Nitab4.5_0002377g0030	ERF012	-2.66	-1.06	-1.16	-3.18	-2.60	-1.86	-1.45	-2.71	GO:0005622//intracellular; GO:0003700//transcription factor activity; GO:0005488//binding; GO:0000160//phosphorelay signal transduction system; GO:0006950//response to stress
Nitab4.5_0005061g0040	ERF025	-2.51	-1.59	-1.63	-3.69	-2.55	-1.78	-1.89	-2.70	GO:0003700//transcription factor activity, sequence-specific DNA binding; GO:0006355//regulation of transcription, DNA-templated
Nitab4.5_0002493g0050	ALMT2	-2.21	-2.57	-2.36	-6.24	-2.50	-2.83	-2.85	-5.30	GO:0005886//plasma membrane; GO:0022857//transmembrane transporter activity; GO:0015743//malate transport; GO:0055085//transmembrane transport
Nitab4.5_0001675g0050	XTH16	-2.20	-1.73	-1.44	-5.10	-2.71	-2.75	-1.75	-3.87	GO:0005618//cell wall; GO:0016762//xyloglucosyl transferase activity; GO:0005975//carbohydrate metabolic process
Nitab4.5_0000446g0040	XTH16	-1.83	-1.26	-2.57	-5.97	-2.14	-2.55	-2.33	-5.29	GO:0005618//cell wall; GO:0016762//xyloglucosyl transferase activity; GO:0005975//carbohydrate metabolic process
Nitab4.5_0000158g0100	ALMT2	-1.83	-1.42	-2.48	-3.93	-2.43	-3.10	-3.52	-5.11	GO:0005886//plasma membrane; GO:0022857//transmembrane transporter activity; GO:0015743//malate transport; GO:0055085//transmembrane transport
Nitab4.5_0004777g0030	YUC9	-1.73	-2.09	-1.04	-5.91	-1.82	-2.74	-2.10	-4.24	GO:0004499//N-dimethylaniline monooxygenase activity; GO:0004791//thioredoxin-disulfide reductase activity; GO:0015036//disulfide oxidoreductase activity
Nitab4.5_0000446g0010	XTH16	-1.57	-2.04	-1.93	-5.58	-2.20	-2.84	-2.08	-4.68	GO:0005618//cell wall; GO:0016762//xyloglucosyl transferase activity; GO:0005975//carbohydrate metabolic process
Nitab4.5_0003734g0040	XTH22	-1.34	-1.18	-2.62	-7.36	-1.45	-2.20	-2.011	-4.74	GO:0004553//hydrolase activity, hydrolyzing O-glycosyl compounds; GO:0005975//carbohydrate metabolic process

### KEGG enrichment analysis of DEGs

**Fig. 5:**
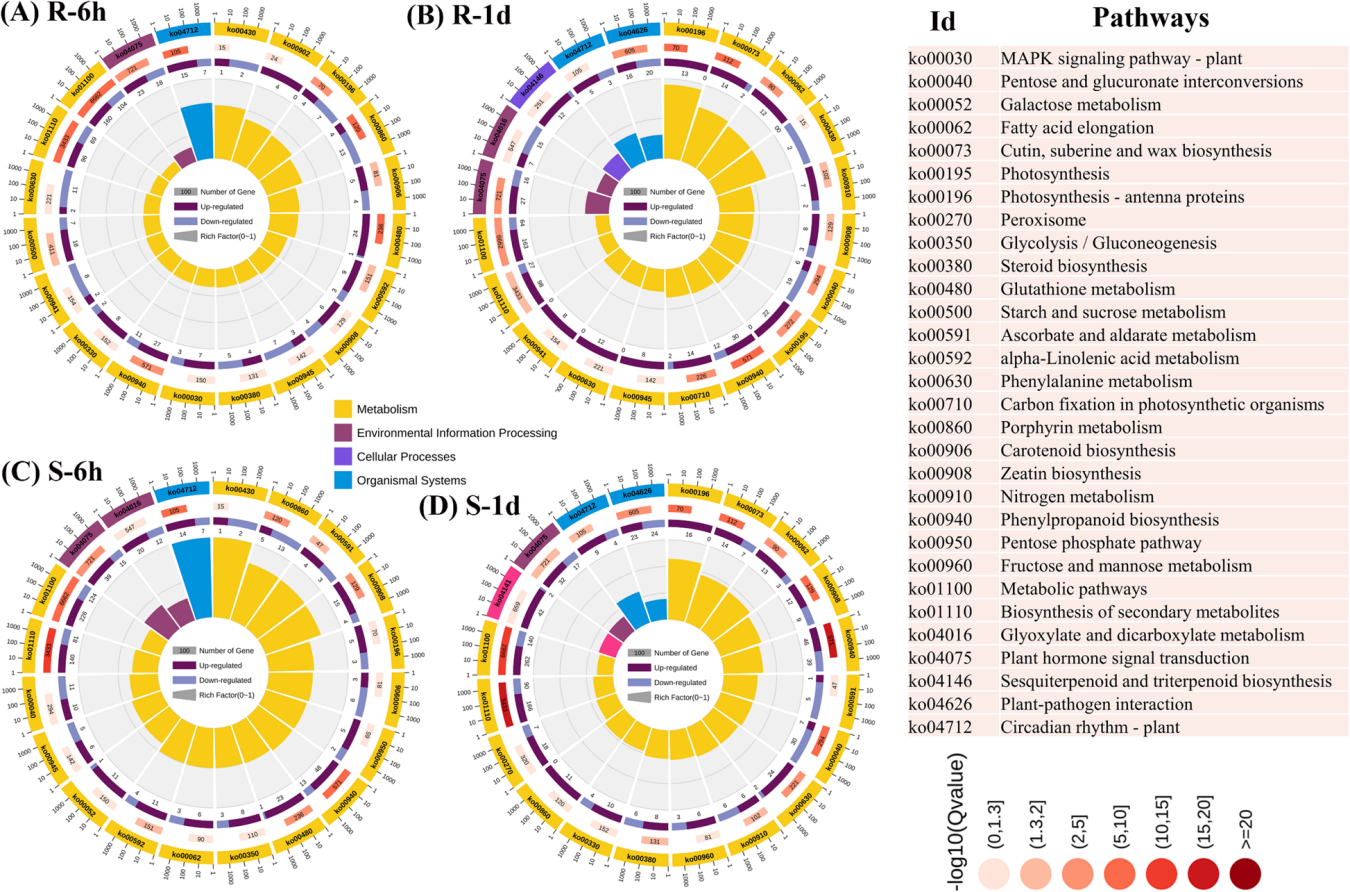
KEGG analysis of differentially expressed genes in bacterial wilt resistant (R) and susceptible (S) tobacco cultivars at 6 h and 1 d. The top KEGG pathways are listed at R-6 h (**A**), R-1d (**B**), S-6 h (**C**), and S-1d (**D**) at *P* ≤ 0.05. The circular diagram represents the enrichment KEGG pathways with the number of up- and down-regulated genes. The outer circular line indicates the pathway ID of each pathway, middle circular line indicates the q-value of enrichment with total number of genes, and inner circular lines indicate the up-regulated (purple) and down-regulated (blue) number of genes

Compared with the control, the DEGs at 6 h, 1 d, 3 d, and 7 d were significantly assigned to 11, 13, 17, and 35 pathways in the R cultivar (Fig. 5A-B and Additional file 1: Table S4) and 10, 14, 19, and 40 pathways in the S cultivar (Fig. 5C-D and Additional file 2: Fig. S8-S9), respectively. In the R cultivar, the highest number of DEGs were enriched in phenylpropanoid biosynthesis, pyruvate metabolism, plant-pathogen interaction, phenylalanine metabolism, carbon metabolism, plant hormone signal transduction, glutathione metabolism, MAPK signaling pathway, steroid biosynthesis, peroxisome, and circadian rhythm, involved in response to *R. solanacearum* at all time-points (Fig. 5A-B and Additional file 1: Table S4). Similarly, biosynthesis of various antibiotics, ribosomes, biosynthesis of amino acids, glycolysis/gluconeogenesis, plant hormone signal transduction, glutathione metabolism, phenylpropanoid biosynthesis, photosynthesis, plant-pathogen interaction, and phenylalanine metabolism were involved in the response of the S cultivar to BW infection at all time-points (Additional file 2: Fig. S8-S9 and Additional file 1: Table S4). KEGG analysis revealed the enrichment of several pathways, including MAPK signaling, phenylpropanoid biosynthesis, plant hormone signal transduction, and phenylalanine and glutathione metabolism in both R and S cultivars (Fig. 5). Additionally, a substantial number of highly up-regulated genes in these cultivars were associated with phenylalanine metabolism, which serves as a precursor for various secondary metabolites in plants.

### Identification of potential candidate genes associated with bacterial wilt resistance

**Fig. 6:**
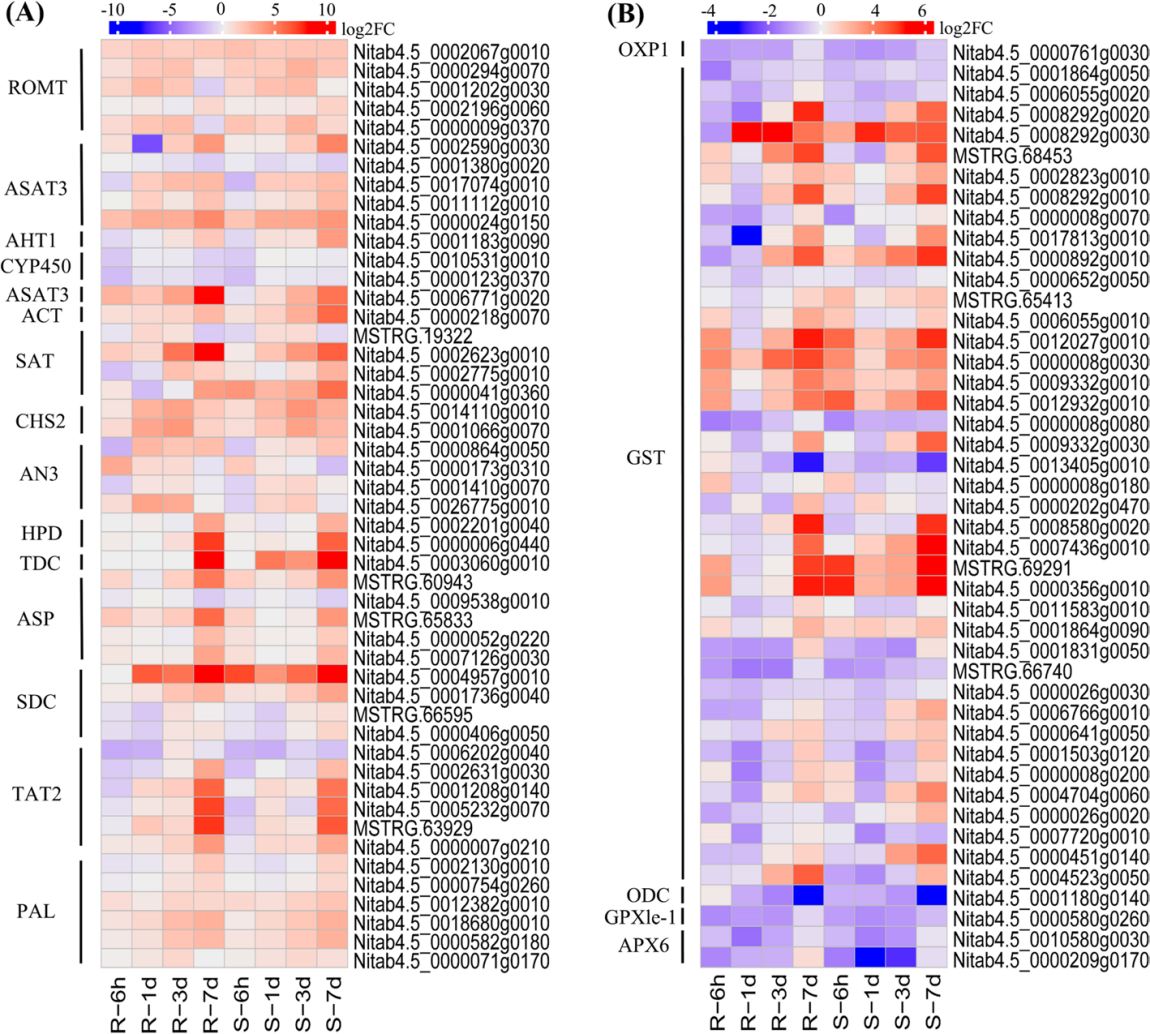
Heatmaps of differentially expressed genes enriched in glutathione and phenylpropane pathways involved in bacterial wilt resistance in tobacco. **A** Number of DEGs involved in the phenylpropane pathway; **B** Number of DEGs involved in glutathione pathway and showed different expression levels at each time point

**Fig. 7:**
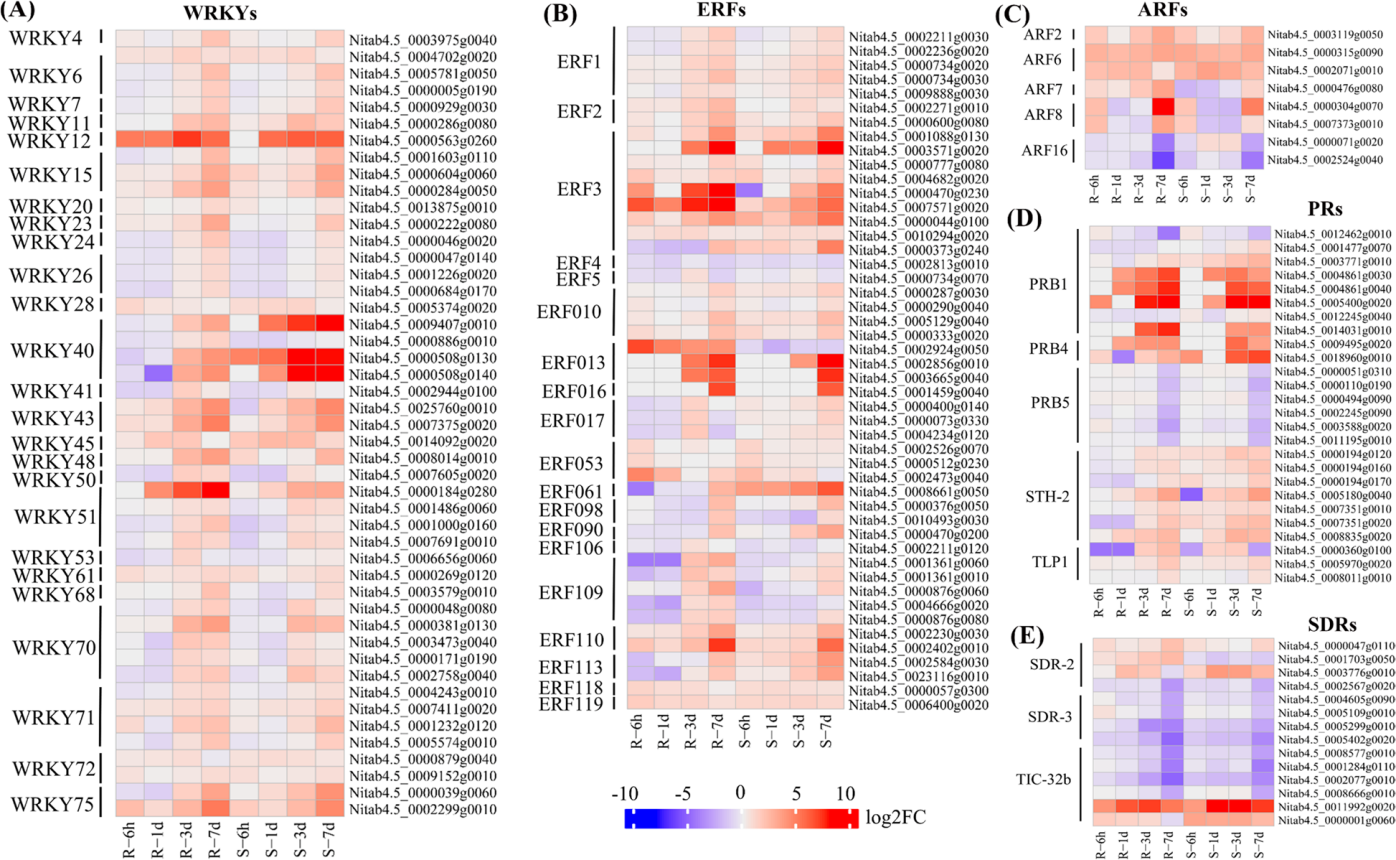
Heatmaps of transcription factors and gene families involved in bacterial wilt resistance in tobacco. The small Fig represent the significant DEGs of (**A**) WRKYs, (**B**) ERFs, (**C**) ARFs, (**D**) PRs, and (**E**) SDRs in both cultivars at different time points after the inoculation of bacterial wilt

Based on GO and KEGG enrichment and functional annotation analysis, we evaluated potential candidate genes related to *R. solanacearum* resistance (Figs. 6 and 7 and Additional file 1: Tables S5–S7), including those involved in phenylalanine metabolism (stilbenoid, diarylheptanoid, and gingerol biosynthesis//ko00945 and flavonoid biosynthesis//ko00941) and glutathione metabolism pathways, and TFs (WRKYs, ARFs, and ERFs) and gene families (SDR, NBS-LR, PRs, and CKX). Hence, DEGs within these pathways or TFs were selected as potential candidate genes for subsequent investigations.

### Pathway-enriched genes for TBW resistance

Forty-nine DEGs involved in the phenylpropane pathway (e.g., flavonoid biosynthesis, stilbenoid, diarylheptanoid, and gingerol biosynthesis) were identified (Fig. 6A and Additional file 1: Table S5), with most up-regulated at 3 d and 7 d. For example, *Nitab4.5_0017074g0010* and *Nitab4.5_0000024g0150* encoding acyl-sugar acyltransferase 3-like were up-regulated at all time points. At the same time, the R cultivar had higher expression levels of three genes (*Nitab4.5_0002623g0010*, *Nitab4.5_0002775g0010*, and *Nitab4.5_0000041g0360*) that encode vinorine synthase-like protein than the S cultivar (Fig. 6A). Specifically, at 6 h after infection, the R and S cultivars had 8 and 4 up-regulated genes, respectively (Fig. 6A). Additionally, the phenylpropane pathway was enriched in up-regulated genes in both cultivars post-infection at different time points. In the R cultivar, 12, 18, and 35 up-regulated genes were enriched at 1 d, 3 d, and 7 d post-infection, respectively, whereas the S cultivar had 4, 16, and 27 up-regulated genes enriched at the same time-points (Fig. 6A). These genes related to cytochrome P450 (CYP), flavanone 3-hydroxylase (AN3), 4-hydroxyphenylpyruvate dioxygenase (HPD), 4-hydroxyphenylpyruvate dioxygenase (HPD), aromatic-L-amino-acid decarboxylase-like (TDC1), aspartate aminotransferase 1, partial (ASP), and phenylalanine ammonia-lyase G4-like (PAL), enhancing plant resistance against *R. solanacearum* (Fig. 6A and Additional file 1: Table S5). Moreover, in the glutathione metabolic pathway, 45 genes encoding 5-oxoprolinase-like (OXP), glutathione S-transferase (GST), ornithine decarboxylase-like (ODC), and L-ascorbate peroxidase 6 (APX), had higher expression at all time-points except 6 h (Fig. 6B). Specifically, the R cultivar up-regulated more genes than the S cultivar at 3d and 7 d post-infection (23 and 43, respectively) (Fig. 6B).

### Transcription factor genes for TBW resistance

WRKY, ARF, and ERF transcription factors are essential regulators of plant resistance [17]. Among them, the WRKY family is the most well-known and plays a vital role in modulating the transcription of resistance-related genes and regulating various plant defense processes. In this study, 47 WRKY genes were significantly expressed in both cultivars at 6 h, 1 d, 3 d, and 7 d (Fig. 7A and Additional file 1: Table S6). Among these, 19 WRKY genes (*WRKY70, WRKY75, WRKY11, WRKY15, WRKY23, WRKY40, WRKY43, WRKY51, WRKY61, WRKY26*, and *WRKY50* family genes) were simultaneously up-regulated in R and S cultivar at 3 and 7, and 14 WRKY genes (*WRKY20, WRKY24, WRKY26, WRKY40, WRKY51, WRKY71, WRKY72, WRKY6, WRKY12, WRKY28, WRKY41*, and *WRKY53* family genes) were only up-regulated in R cultivar at 7d (Fig. 7A). Notably, four genes, *Nitab4.5_0007605g0020* (*WRKY50*), *Nitab4.5_0001000g0160* (*WRKY51*), *Nitab4.5_0000048g0080* (*WRKY70*), and *Nitab4.5_0014092g0020* (*WRKY45*) were up-regulated in R cultivars at all time-points except 7d (Fig. 7A and Additional file 1: Table S6). It is speculated that three genes (*Nitab4.5_0004702g0020, Nitab4.5_0005781g0050*, and *Nitab4.5_0000005g0190*) of *WRKY6* TFs were identified at R-6 h and S-6 h with a high expression that could act as positive regulators of TBW resistance (Fig. 7A).

Moreover, ERFs have essential biological functions in various life activities, including plant growth, development, and environmental response. We identified 48 ERFs DEGs in both cultivars (Fig. 7B and Additional file 1: Table S6), of which 5 (*ERF1*), 2 (*ERF2*), 9 (*ERF3*), *Nitab4.5_0002813g0010* (*ERF4*), *Nitab4.5_0000734g0070* (*ERF5*), 4 (*ERF10*), and 5 (*ERF109*) family genes were up-regulated at least one-time point in R and S cultivars (Fig. 7B). Like, *Nitab4.5_0002230g0030* (*ERF110*) and *Nitab4.5_0002236g0020* (*ERF1*), genes were up-regulated in R cultivar at 1d, 3d, and 7d while these genes only expressed in S cultivar at 3d after infection (Fig. 7B and Additional file 1: Table S6). Moreover, one gene, *Nitab4.5_0006400g0020* (*ERF119*), was up-regulated in both cultivars at all time points, whereas *Nitab4.5_0002271g0010* (*ERF2*), *Nitab4.5_0000734g0070* (*ERF5*), *Nitab4.5_0002924g0050* (*ERF13*), and *Nitab4.5_0001459g0040* (*ERF016*), were up-regulated only in R cultivar (Fig. 7B). In addition, eight auxin response factor (ARFs) DEGs were identified in response to BW in tobacco R and S cultivars (Fig. 7C and Additional file 1: Table S6). Three genes, *Nitab4.5_0003119g0050* (*ARF2*), *Nitab4.5_0000315g0090* (*ARF6*), and *Nitab4.5_0002071g0010* (*ARF6*), were highly expressed in the early stage of infection (Fig. 7C). Two genes, *Nitab4.5_0000476g0080* (*ARF7*) and *Nitab4.5_0007373g0010* (*ARF8*), were up-regulated only in Resistant cultivars.

In addition, we identified TFs that were Differentially expressed at 6 h of infection in the R and S cultivars; for example, *Nitab4.5_0000315g0090* (*ARF6*) was up-regulated at 6 h in both cultivars, while *Nitab4.5_0002071g0010* (*ARF6*) was only up-regulated at 6 h in S cultivar. Similarly, ten WRKY genes were highly expressed at 6 h; among these, three DEGs (*WRKY61, WRKY75*, and *WRKY28*) were only identified in R cultivar, whereas seven genes, including *WRKY40, WRKY50, WRKY15, WRKY45*, and *WRKY51* were identified in S cultivar. Seven ERFs, *Nitab4.5_0002526g0070* (*ERF053*), *Nitab4.5_0000512g0230* (*ERF053*), *Nitab4.5_0002473g0040* (*ERF053*), *Nitab4.5_0004682g0020* (*ERF.C.3*), *Nitab4.5_0000333g0020* (*ERF010*), *Nitab4.5_0000057g0300* (*ERF118*), and *Nitab4.5_0006400g0020* (*ERF119*), were high up-regulated at 6 h in both cultivars, and other four (*ERF106, ERF109, ERF110*, and *ERF.C.3*), were down-regulated in S cultivar. These genes might be considered as primary responses of the plant.

### Gene families involve in TBW resistance

NBS-LRR genes encode nucleotide-binding site leucine-rich repeat (NBS-LRR) proteins involved in pathogen recognition and signaling. We identified two novel genes (*MSTRG.61386-R1B-17* and *MSTRG.61568*) up-regulated in both cultivars at 7 d (Additional file 1: Table S7). Further, 26 genes that encode pathogenesis-related (PRs) proteins were identified in both cultivars, which may be involved in various aspects of the plant defense response (Fig. 7D and Additional file 1: Table S7). Among these, *Nitab4.5_0014031g0010* (*PRB1*), *Nitab4.5_0004861g0030* (*PRB1*), *Nitab4.5_0008835g0020* (*STH-2*), and *Nitab4.5_0000360g0100* (*TLP1*) were highly differentially expressed in the R cultivar than the S cultivar at 1 d, 3 d, and 7 d (Fig. 7D). Additionally, *PRB5* genes were down-regulated in both cultivars. In this study, 14 DEGs encode short-chain dehydrogenase/reductase—detected at different time points in the R and S cultivars after bacterial infection—involved in the biosynthesis of phytohormones that play a key function in defense response of plants against pathogens and BW (Fig. 7E and Additional file 1: Table S7). *Nitab4.5_0011992g0020* and *Nitab4.5_0000001g0060* were significantly up-regulated in both cultivars at all time points. One gene, *Nitab4.5_0001703g0050* (*SDR2a*), was highly expressed in the R cultivar but low in the S cultivar (Fig. 7E). The remaining SDR genes showed an initial up-regulation during the early stage of infection, followed by a down-regulation after post-infection.

### Validation of transcriptomic data through quantitative real-time PCR

**Fig. 8:**
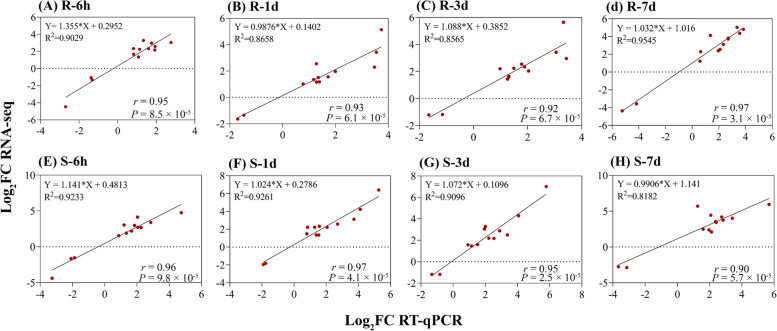
Validation of 13 DEGs in response to bacterial wilt resistance via RT-qPCR. The RT-qPCR and RNA-seq log_2_FC values were compared using correlation analysis at R-6 h (**A**), R-1d (**B**), R-3d (**C**), R-7d (**D**), S-6 h (**E**), S-1d (**F**), S-3d (**G**), and S-7d (**H**). R and P indicate the correlation coefficient and corresponding *P-value*. The correlation showed a significant positive relationship between the RT-qPCR and RNA-seq at each time point in R and S cultivars

To measure the expression levels of potential resistance-related genes, 13 DEGs (11 up-regulated and 2 down-regulated) were randomly selected and analyzed by qRT-PCR at 6 h, 1 d, 3 d, and 7 d after infection. Of these, two genes (*Nitab4.5_0000356g0010* and *Nitab4.5_0012932g0010*) associated with GST, one gene (*Nitab4.5_0005779g0060*) related to CKX5, and one gene (*Nitab4.5_0008666g0010*) related to short-chain dehydrogenase TIC32. As a result, the RT-qPCR produced similar results as the RNA-seq data at 6 h, 1 d, 3 d, and 7 d (Fig. 8A-H). The correlation analysis revealed a highly significant positive correlation between RT-qPCR and RNA-seq data at R-6 h (*r* = 0.95 and *P* = 8.5 × 10^–5^) and S-6 h (*r* = 0.96 and *P* = 9.8 × 10^–5^), indicating methodological reliability and supports the consistency of the RNA-sequencing data (Fig. 8A-H).

## Discussion

Tobacco bacterial wilt caused by *R. solanacearum* is a major soil-borne disease that adversely affects the quality and yield of tobacco crops. The survival of *R. solanacearum* in non-host or soil, and its ability to cause severe damage once the host appears makes TBW a significant economic threat [7, 9, 17, 44, 45]. The plant's transcriptional response to pathogen infection is a dynamic process involving the modulation of signals and compounds that change over time [29, 33, 46]. This study identified 239 potential candidate genes associated with the phenylpropane pathway, glutathione metabolism, WRKY, ERFs, ARFs, PRs, and NBS-LRR using root transcriptomic sequencing data of R and S cultivars at 6 h, 1 d, 3 d, and 7 d (Fig. 6–7 and Additional file 1: Tables S5–S7).

The Illumina sequencing data was reliable and accurately verified using RT-qPCR expression (Fig. 8) with a high Q30 base rate ranging from 92.78–94.94%. A total of 81,534 genes were identified, with 69,500 known and 12,034 novel genes, in line with published studies [33, 46]. The results indicate that BW infection significantly changes gene expression in R and S cultivars, with 20,711 DEGs in the R cultivar and 16,663 DEGs in the S cultivar (Fig. 3A-B and Additional file 1: Table S2). Among these, 554 were common between all samples (Fig. 3B-C), suggesting that the response to BW infection is cultivar-specific and occurs at different time-points in the two cultivars, similar to studies on tobacco [9], tomato [37], pepper [34], and *Arabidopsis* [47]. For example, Ishihara et al. [32] identified 140 DEGs in a resistant tomato variety and none in a susceptible variety.

The identified DEGs were involved in 1,236 and 1,121 GO terms in the R and S cultivars, respectively (Fig. 4 and Additional file 1: Table S3). The R cultivar had a stronger defense response to *R. solanacearum* infection than the S cultivar, as evidenced by the up-regulation of genes involved in transferase activity, cell wall organization, hormone signal transduction, abiotic stress response, and oxidative stress response. In contrast, the S cultivar had a weaker defense response, as evidenced by the up-regulation of genes involved in tetrapyrrole binding, oxidoreductase activity, and carbohydrate metabolic processes (Fig. 4 and Additional file 1: Table S3). Our results are consistent with previous studies on BW resistance in solanaceous crops, linking resistance to increased expression of genes related to defense mechanisms, cell wall organization, and oxidative stress response [29, 32–34, 48]. For example, Pan et al. [33] found that 3,967 DEGs were associated with endocytosis, cell wall, signal transduction, and starch/sucrose metabolism in response to BW infection.

The KEGG analysis showed that the DEGs at 6 h, 1 d, 3 d, and 7 d were significantly assigned to different pathways in the R and S cultivars (Fig. 5 and Additional file 1: Table S4). The highest up-regulated genes in the R cultivar (Additional file 2: Fig. S6-S7 and Additional file 1: Table S4) were enriched in plant hormone signal transduction, glutathione metabolism, phenylpropanoid biosynthesis, plant–pathogen interactions, and MAPK signaling pathway, consistent with studies investigating the molecular mechanisms of BW resistance in tomato [37], eggplant [36], tobacco [17, 30, 33], and potato [8, 35]. Furthermore, other studies reported the involvement of the enriched pathways in tobacco defense against *R. solanacearum* [26, 29, 46]. These results also align with studies that reported the activation of multiple defense mechanisms in response to TBW infection in resistant tomato and eggplant cultivars, including the activation of glutathione metabolism and phenylpropanoid biosynthesis [32, 34, 49].

The present study identified 49 potential candidate genes in the phenylpropanoid pathway (Fig. 6A). Activation of the phenylpropanoid pathway has been associated with increased resistance to BW in tobacco [9, 35, 46]. Six PAL genes (*Nitab4.5_0002130g0010, Nitab4.5_0000754g0260, Nitab4.5_0012382g0010, Nitab4.5_0018680g0010, Nitab4.5_0000582g0180*, and *Nitab4.5_0000071g0170*) were up-regulated at 3 d and 7 d post-infection (Fig. 6A). Several studies have investigated the role of the phenylpropanoid pathway in defending against BW [17, 35, 37]. For example, Park et al. [50] reported that the up-regulation of PAL genes plays a significant role in the plant defense response to BW, while the *OsPAL2* mutant gene in rice contributed to the control of pathogen disease resistance [51]. This study revealed two genes (*CYP98A2* and *CYP98A3*) of cytochrome CYP450 (Fig. 6A) that play an essential role in BW resistance in many crops [52–55]. Moreover, we identified 46 candidate genes involved in glutathione metabolism, including *APX, GST, ODC, OXP*, and *PRP* (Fig. 6B), which play a crucial role in the detoxification of ROS and the regulation of cellular signaling pathways that lead to the activation of defense mechanisms [6, 30, 37, 56, 57]. Our results showed that the R cultivar up-regulated more genes involved in glutathione metabolism than the S cultivar at 3 d and 7 d after TBW inoculation (Fig. 6B), consistent with other studies [7, 36, 58]. Overexpression of *GST* and *GPX* genes increased expression activity twofold in wild-type tobacco seedlings, contributing to chilling or salt stress tolerance [59]. Li et al. [17] reported up-regulated DEGs involved in the phenylpropanoid pathway and glutathione metabolism for TBW resistance. Therefore, the glutathione metabolism and phenylpropanoid pathway are essential defense mechanisms in tobacco plants against BW caused by *R. solanacearum*. Further research is needed to understand how these pathways contribute to disease resistance in tobacco.

**Fig. 9:**
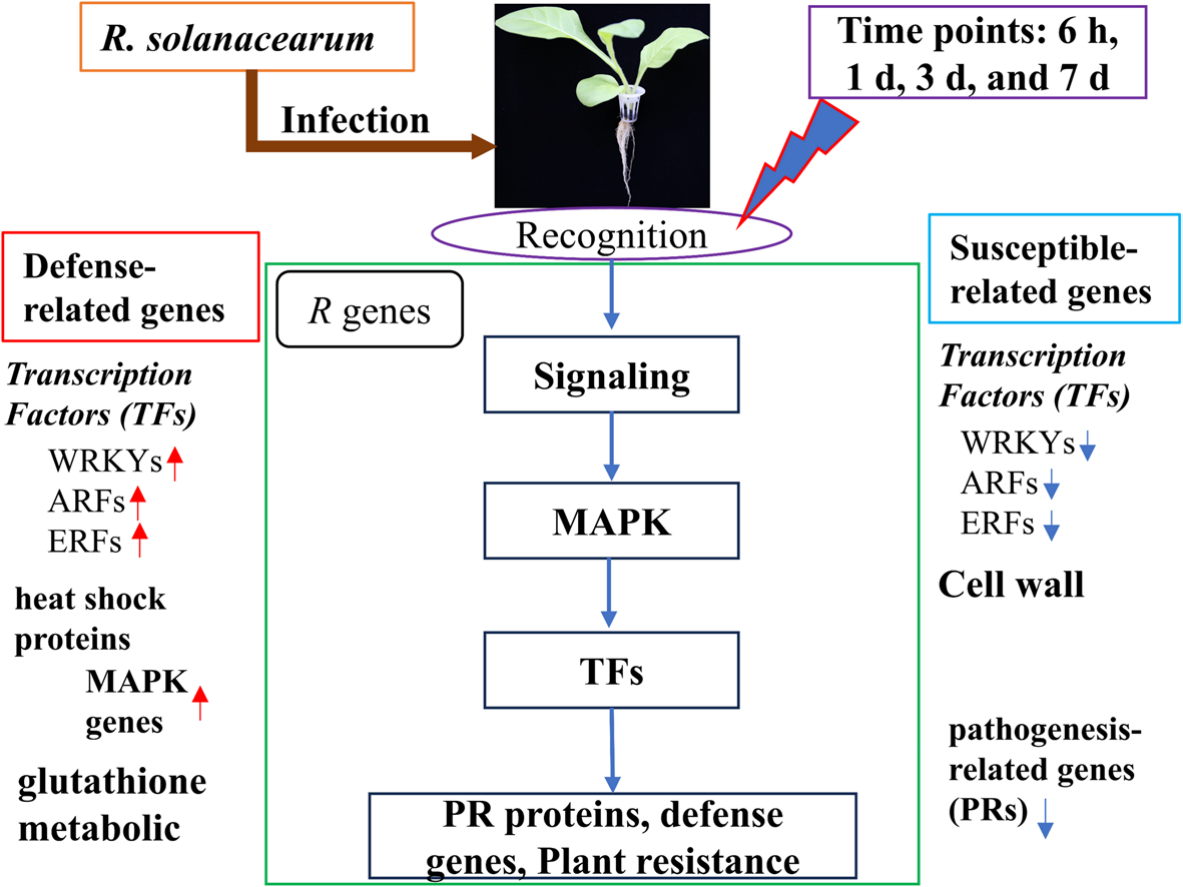
A model pathway involved in resistance against tobacco bacterial wilt. The tobacco reaction to *R. solanacearum* infection and the systematic pathway involved in resistance. The signals from the pathogens were recognized through related receptors (R genes) and transcription factors, which triggered the plant defense mechanism. After that, defense-related molecules/genes, including PR-proteins, metabolites, glutathione and phenylpropane genes, were activated to cause resistance to pathogens through high expression levels

TFs are crucial plant regulators [38, 60], with WRKY TFs playing essential roles in plant immunity against various stresses. *WRKY40*, *WRKY6*, *WRKY27*, and *WRKY22* have been identified as positive regulators of Solanaceae crop resistance to BW in several [38, 60–62]. In this study, *WRKY6* (*Nitab4.5_0004702g0020, Nitab4.5_0005781g0050*, and *Nitab4.5_0000005g0190*) and *WRKY11* (*Nitab4.5_0000286g0080*) were found to exhibit a resistance response to TBW in the R cultivar (Fig. 7A), particularly in the later stage, consistent with the response pattern observed in pepper to BW [62]. These findings indicate that these genes may be important regulators of tobacco resistance against BW [63, 64]. Moreover, ERFs are a class of plant TFs involved in biological and abiotic stress responses [17, 65, 66]. In this study, 48 ERFs genes were significantly expressed in R and S cultivars at all time points (Fig. 7B). The expression pattern of ERF5 (*Nitab4.5_0000734g0070*) in the R cultivar was distinct from that in the S cultivar (Fig. 7B), especially at 3 d. The eight ARF genes identified in the present study were up-regulated at 3 d and 7 d after infection (Fig. C); these genes play a critical role in regulating plant growth and development to improve plant defense mechanisms against pathogens [17, 33, 39]. Pathogenesis-related proteins (PRs) are essential for BW resistance [67, 68], with the present study identifying 26 PR genes (*PR-1*, *PR-4*, and *PR-5*) in both cultivars at all time points (Fig. 7D). Two *PR-1* genes (*Nitab4.5_0004861g0040* and *Nitab4.5_0005400g0020*) were up-regulated at 3 d and 7 d in both cultivars (Fig. 7D), similar to previous studies [68, 69]. Rivière et al. [69] found that *PR-1b* was up-regulated in tobacco plants resistant to BW and that silencing *PR-1b* increased disease susceptibility. Similarly, Alamillo et al. [70] and Pruss et al. [71]reported up-regulated expression of *PR-2* in tobacco infected with *R. solanacearum*, while silencing *PR-2* increased disease susceptibility [72]. Further, we identified 14 SDR genes involved in various metabolic pathways, including detoxification and hormone biosynthesis (Fig. 7E). In tobacco, SDR genes play a role in the defense response against bacterial pathogens: Yu et al. [73] reported that SDR genes are involved in the biosynthesis of jasmonic acid, which is critical for defense response against pathogens. Overall, the present study identified 239 potential candidate genes associated with pathways, TFs, and gene families (Figs. 6 and 7 and Additional file 1: Tables S5–S7), which will help understand the defense response mechanism and could be potential targets for improving resistance to BW disease. In summary, our results revealed that DEGs associated with phenylpropane/flavonoids pathway, glutathione metabolic pathway, WRKY, ERFs, ARFs, pathogenesis-related genes (PRs), and short-chain dehydrogenase/reductase genes essential to control the resistance and programmed cell death. Of these, most of the defense-related genes were up-regulated in the R cultivar compared to the S cultivar at an early stage of infection. Therefore, our hypothesized model indicates the involvement of defense-related and susceptible-related genes networking of R. solanacearum in the tobacco system (Fig. 9).

## Conclusion

This study used high throughput sequencing to identify DEGs and related pathways in resistant and susceptible cultivars under R. solanacearum infection at early and late seedling stages. In early plant response after 6 h, 2 ARFs, 10 WRKY, and 11 ERFs genes were differentially expressed, which might be the plants' primary response. The R and S cultivars had similar response levels at the early stage, but the R cultivar-related DEGs had specific expression patterns in response to infection after 7 d. Finally, 239 potential candidate genes were identified using GO, KEGG, functional enrichment analysis, and literature search. These DEGs in the resistant cultivar are related to glutathione metabolism and phenylpropanoid biosynthesis, TFs, and PRs, and are likely the main substances conferring root resistance against BW infection in tobacco. Additionally, two novel genes (MSTRG.61386-R1B-17 and MSTRG.61568) were up-regulated in both cultivars at 7 d, which can be used for functional studies to find the exact role in BW resistance. The finding of this study shows the molecular mechanisms involved in tobacco resistance to bacterial wilt and provides an important source for controlling and breeding tobacco plants resistant to TBW.

## Methods

### Plant material and growth conditions

Two tobacco cultivars, D101 (R) and Honghuadajinyuan (S), were received from Nanxiong Scientific Research Institute of Guangdong Research Institute of Tobacco Science, Shaoguan, China. The experiments were conducted in the greenhouse of Guangzhou University, China. Seeds of both cultivars were surface-sterilized with distilled water and spread on a black plastic tray with white and pit seedling sponges. The tray was covered with a layer of fresh-keeping film and placed in the growth chamber to germinate at 28℃, 60% relative humidity, and 16 h/8 h light/dark photoperiod. After germination, seedlings in the crosswise stage were transferred into hydroponic boxes (41 × 24 × 14 cm; length × width × height). Hoagland’s nutrient solution was added to the aerated hydroponic containers, comprising 945 mg/L Ca(NO_3_)_2_·4H_2_O, 607 mg/L KNO_3_, 115 mg/L (NH_4_)_2_HPO_4_, 493 mg/L MgSO_4_·7H_2_O, 30 mg/L [-CH_2_N(CH_2_COONa)CH_2_COO]_2_Fe, 2.86 mg/L H_3_BO_3_, 2.13 mg/L MnSO_4_.4H_2_O, 0.22 mg/L ZnSO_4_.7H_2_O, 0.08 mg/L CuSO_4_.5H_2_O, and 0.02 mg/L (NH_4_)_2_MoO_4_.

### Inoculation treatment and sample collection

The highly pathogenic *R. solanacearum* strain HBLC5 was isolated from tobacco plants obtained from Hukou experimental station in Nanxiong City as described in our previous studies [7, 15]. For inoculation preparation, the *R. solanacearum* strain HBLC5 was cultured on BG medium and reproduced in Liquid LB medium by shaking at 30℃ and 180 rpm for two days. Five-leaf stage plants were infected with 300 mL cell suspension (adjusted to 1 × 10^8^ CFU/mL and OD_600nm_ = 0.1) by irrigating roots in one pot. The control was treated with sterile water instead of the bacterial solution. Each tobacco variety was grown in three pots, each with 60 seedlings. Tobacco roots from three individual seedlings taken from one pot were considered one biological replicate. Therefore, three biological replicates of each variety were harvested from three pots at every time point. After inoculation, the roots from R and S cultivars were collected at R/S-0 h, R/S-6 h, R/S-1d, R/S-3d, and R/S-7d, and the samples were stored at -80 °C after freezing in liquid nitrogen until RNA extraction.

### RNA extraction, library preparation, and Illumina sequencing

The total RNA of tobacco seedlings roots at five-six leaf stage (~ 30 days) was extracted from three biological replicates at each time-point using the TRIzol Reagent Kit (Invitrogen, Carlsbad, CA, USA) with the manufacturer’s instructions. In short, RNA was extracted from the frozen samples, and DNase I (TakaraBio, Japan) was used to remove DNA contamination. The RNA quality and integrity were assessed through 1% agarose gel electrophoresis and a microplate spectrophotometer (BioTek Company, USA). To develop the cDNA libraries, mRNA with polyA tails was purified using Oligo(dT) magnetic beads and fragmented. The first and second strands of cDNA were synthesized with random hexamer primers and DNA polymerase I, respectively. Adapters were ligated to double-stranded cDNAs, which were then exposed to PCR amplification and purification. The quality of the cDNA libraries was measured using an Agilent 2100 Bioanalyzer, and sequencing was performed on the Illumina Novaseq 6000 platform by Genedenovo Biotechnology Co., Ltd in Guangzhou, China.

### Sequencing data analysis and genomic annotation

The sequenced raw reads were processed using fastp v0.18.0 software [74] to determine the reliability and quality of sequenced data and remove the reads that contained adaptors, ≤ 10% unknown bases, and low-quality reads at Q ≤ 20%. Clean reads were used to determine Q20, Q30, and GC contents and for all further downstream analyses. The Nitab4.5 reference genome of *N. tabacum* [75] and gene annotation files were downloaded from the solgenomics database (https://solgenomics.net/organism/Nicotiana_tabacum/genome). The first step was to align the clean reads (i.e., reads filtered for quality and adapter sequences) to a reference genome. In this case, the clean reads were aligned to Nitab4.5 using HISAT2 v2.1.0 [76] and Bowtie2 v2.2.5 [77]. After alignment, the sequences of all assembled genes were aligned to different databases for functional annotation, including the Nitab4.5 reference genome, UniProt, NT, KEGG, GO, and COG using Trinotate (http://trinotate.github.io/) software with default parameters except E ≤ 1e-5. Then, transcripts were reconstructed using StringTie v2.2.0 [78] based on the HISAT2 alignment. Transcript abundance was measured as FPKM values using RSEM software [79]. Finally, the principal component analysis (PCA) and Pearson’s correlation coefficients were calculated using the R4.1.0 environment (http://www.r-project.org/) based on the FPKM expression data.

### Identification of differentially expressed genes

DESeq2 R package [80] was used to perform differential expression analysis by pairwise comparison of the control and treatments at 6 h, 1 d, 3 d, and 7 d. The *P-value* was adjusted using the Benjamini–Hochberg method with an FDR threshold of 0.05, and genes were identified as up- or down-regulated based on the |log2FC|> 1 criteria. The hierarchical K-mean clustering method was used to analyze the DEGs using the hclust function in R version 4.1.0 (http://www.r-project.org/), and DEGs were then visualized using a heatmap as log_2_FC input.

### GO and KEGG enrichment analysis

GO enrichment analysis of DEGs was performed using the GO database (http://www.geneontology.org/) with a significance threshold of corrected *P* ≤ 0.05. KEGG enrichment analysis was conducted using the online KEGG database (https://www.genome.jp/kegg/kegg1.html) at *P* ≤ 0.05 as a significance threshold to identify significant biological functions and metabolic pathways [81]. The clusterProfiler v3.4.4 software was used to visualize the top GO terms and KEGG pathways [82].

### Gene expression analysis through RT-qPCR

Thirteen genes were randomly selected with different expression profiles to validate sequencing data by RT-qPCR using specific primers presented in Additional file 1: Table S8. Reverse transcription was completed using the HiScript® III RT SuperMix for PCR (+ gDNA wiper) Kit (Vazyme Biotech Co., Ltd). The qRT-PCR was performed using 1 µL cDNA template mixed with 10 µL 2 × ChamQ SYBR qPCR Master Mix (Vazyme Biotech Co., Ltd) and 0.4 µL each of the forward and reverse primers in a final volume of 20 µL. Amplification consisted of 40 cycles of 95℃ for 10 s, 60℃ for 30 s, and 95℃ for 15 s, followed by a denaturation step to generate the melting curves. The *Ntubc2* (accession number: AB026056) was selected as an internal reference gene, and gene expression profiles were measured using the 2-∆∆CT method. Three technical and biological replicates were used for each sample of cultivars at all time points.

### Supplementary Information

**Additional file 1.**

**Additional file 2.**

## Data Availability

The study data sets can be accessed at NCBI database under accession PRJNA935858 (https://www.ncbi.nlm.nih.gov/bioproject/PRJNA935858).

## Abbreviations

*DEGs*: Differentially expressed genes
R: Resistant
S: Suceptible
TF: Transcriptional factors
