# Supplementary material for: Uncovering the transcriptional responses of tobacco (Nicotiana tabacum L.) roots to Ralstonia solanacearum infection: a comparative study of resistant and susceptible cultivars
Source: BMC Plant Biol. 2023 Dec 6;23:620. doi: 10.1186/s12870-023-04633-w (PMC10699017; doi:10.1186/s12870-023-04633-w)
Supplement: Supplementary file 2 — Additional file 2. [file 12870_2023_4633_MOESM2_ESM.docx]

**Supplementary**

**Uncovering the transcriptional responses of tobacco (*Nicotiana tabacum* L.) roots to *Ralstonia solanacearum* infection: A comparative study of resistant and susceptible cultivars**

Hailing Zhang^1, †^, Muhammad Ikram^1, †^, Ronghua Li^1, †^, Yanshi Xia^1^, Weicai Zhao^2^, Qinghua Yuan^3, *^, Kadambot H.M Siddique^4^, Peiguo Guo^1, *^

^1^ Guangdong Provincial Key Laboratory of Plant Adaptation and Molecular Design, School of Life Sciences, Guangzhou University, Guangzhou 510006, China

^2^ Guangdong Research Institute of Tobacco Science, Shaoguan 512029, China

^3^ Guangdong Provincial Engineering & Technology Research Center for Tobacco Breeding and Comprehensive Utilization, Guangdong Key Laboratory for Crops Genetic Improvement, Crops Research Institute, Guangdong Academy of Agricultural Sciences (GAAS), Guangzhou 510640, China

^4^ The UWA Institute of Agriculture, The University of Western Australia, Perth, WA 6001, Australia

† Both authors contributed equally to this work.

* Corresponding author: Qinghua Yuan, [qinghua654321@126.com](mailto:qinghua654321@126.com); Peiguo Guo, email: [guopg@gzhu.edu.cn](mailto:guopg@gzhu.edu.cn)


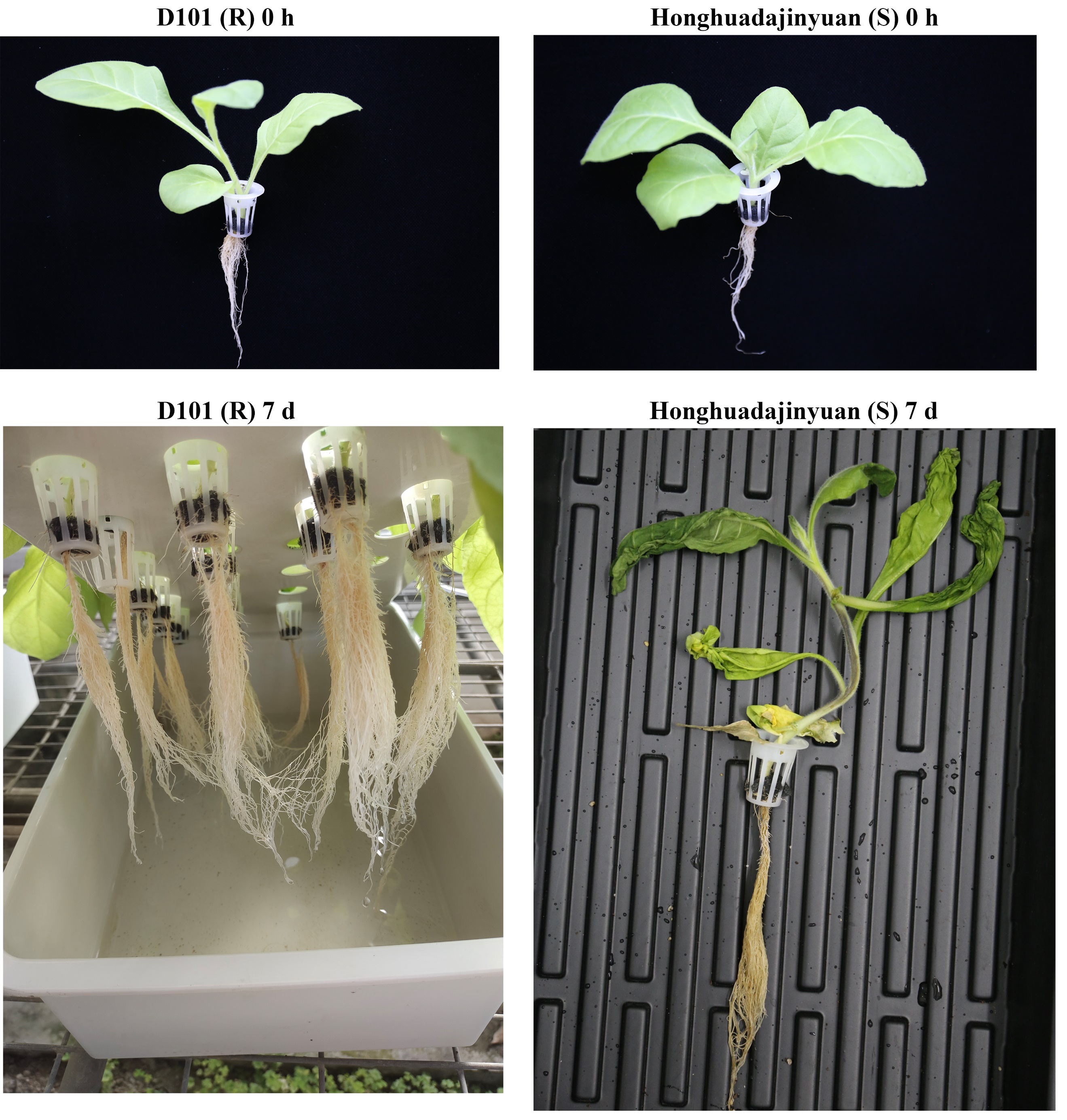


**Fig. S1 The phenotypes of roots of infected and healthy seedlings of Honghuadajinyuan (S) and D101 (R) cultivars**. The upper two small figures show the healthy seedling, and the lower two show infected seedling roots. The roots of infected plants at 7 d had clear symptoms of bacterial wilt.

**
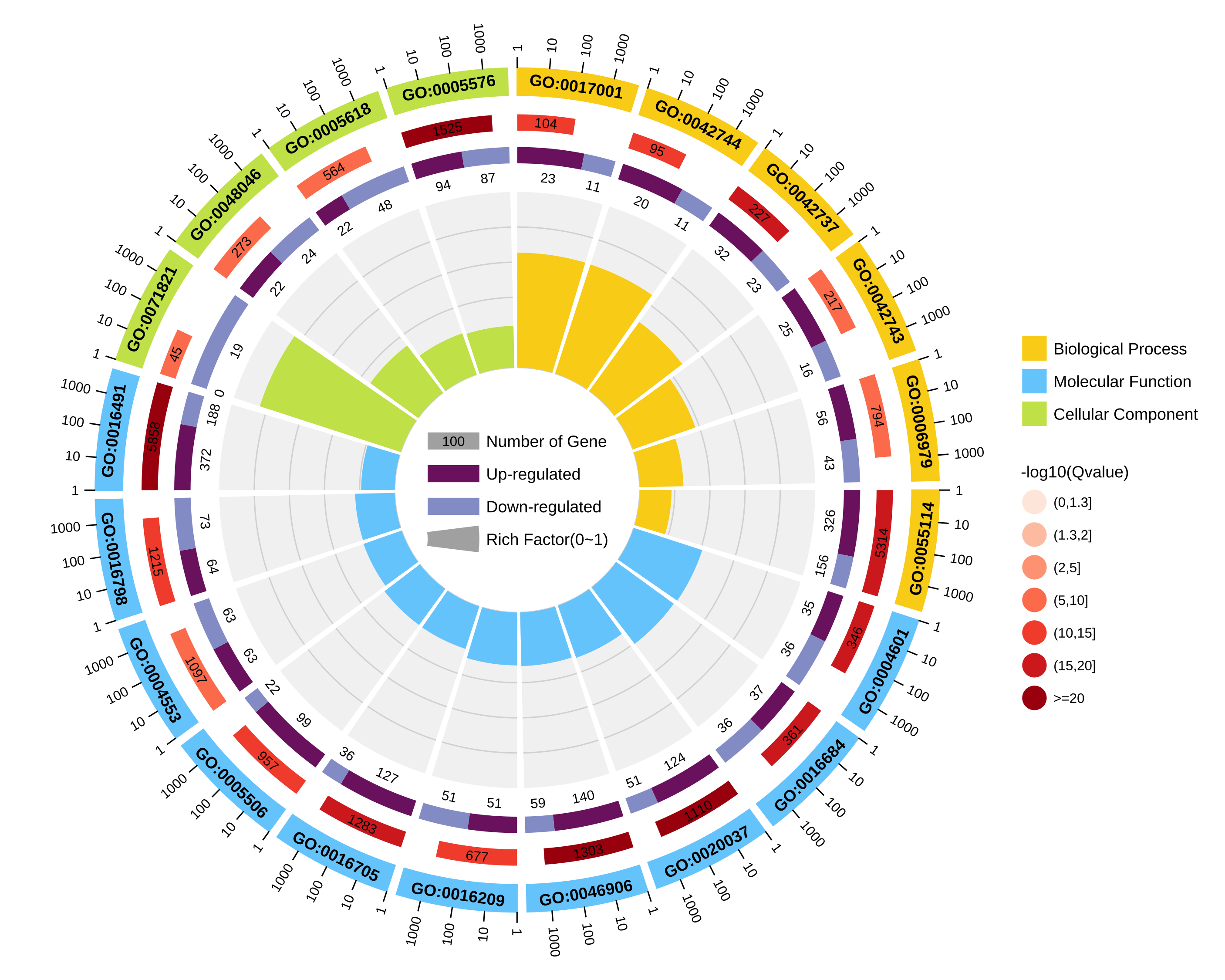
**

**Fig. S2 GO enrichment analysis of up- and down-regulated genes in resistant tobacco cultivar at 3 days after bacterial wilt infection.** The top GO terms in three categories are listed at *P* ≤ 0.05.

**
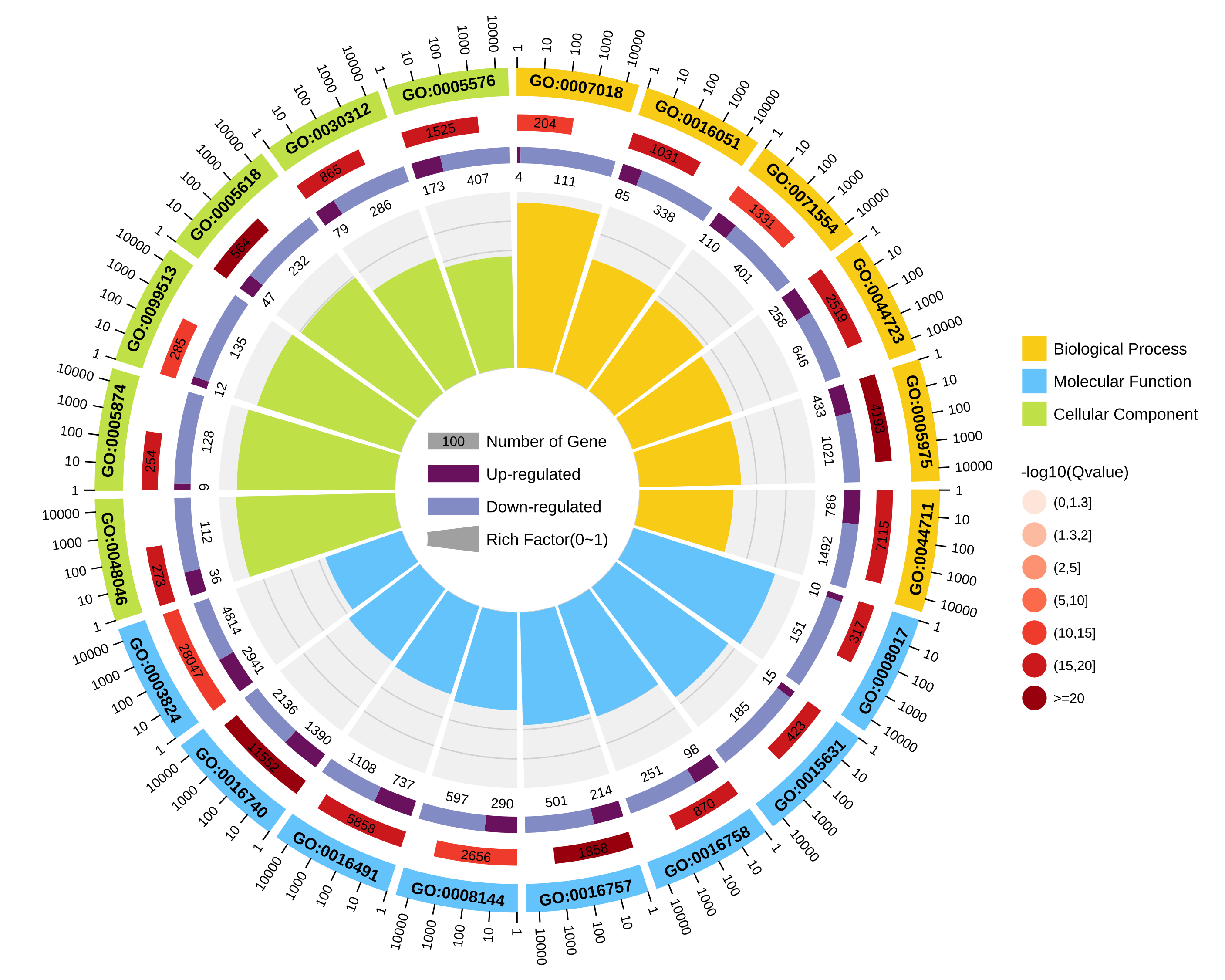
**

**Fig. S3 GO enrichment analysis of up- and down-regulated genes in resistant tobacco cultivar at 7 days after bacterial wilt infection.** The top GO terms in three categories are listed at *P* ≤ 0.05.

**
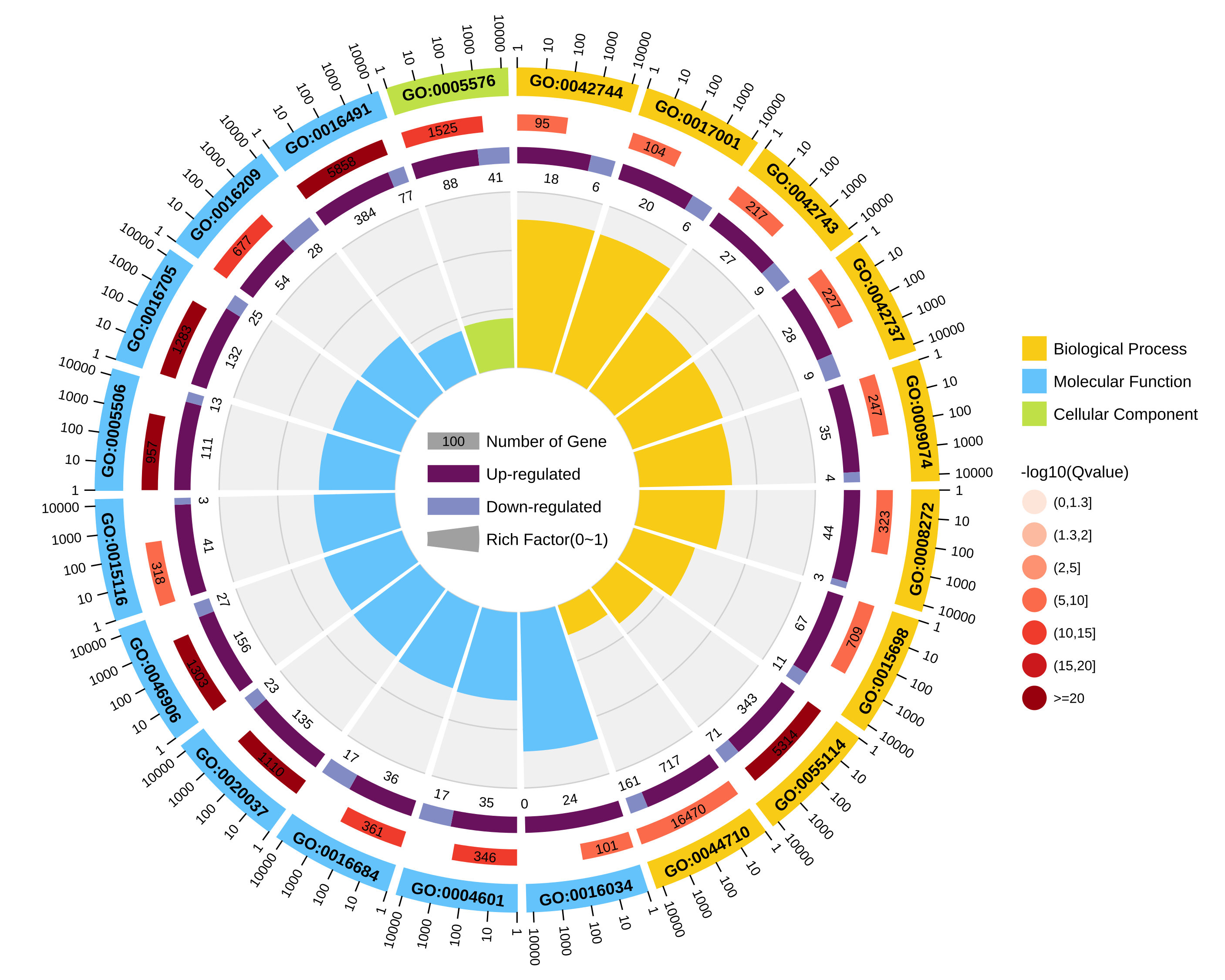
**

**Fig. S4 GO enrichment analysis of up- and down-regulated genes in susceptible tobacco cultivar at 3 days after bacterial wilt infection.** The top GO terms in three categories are listed at *P* ≤ 0.05.

**
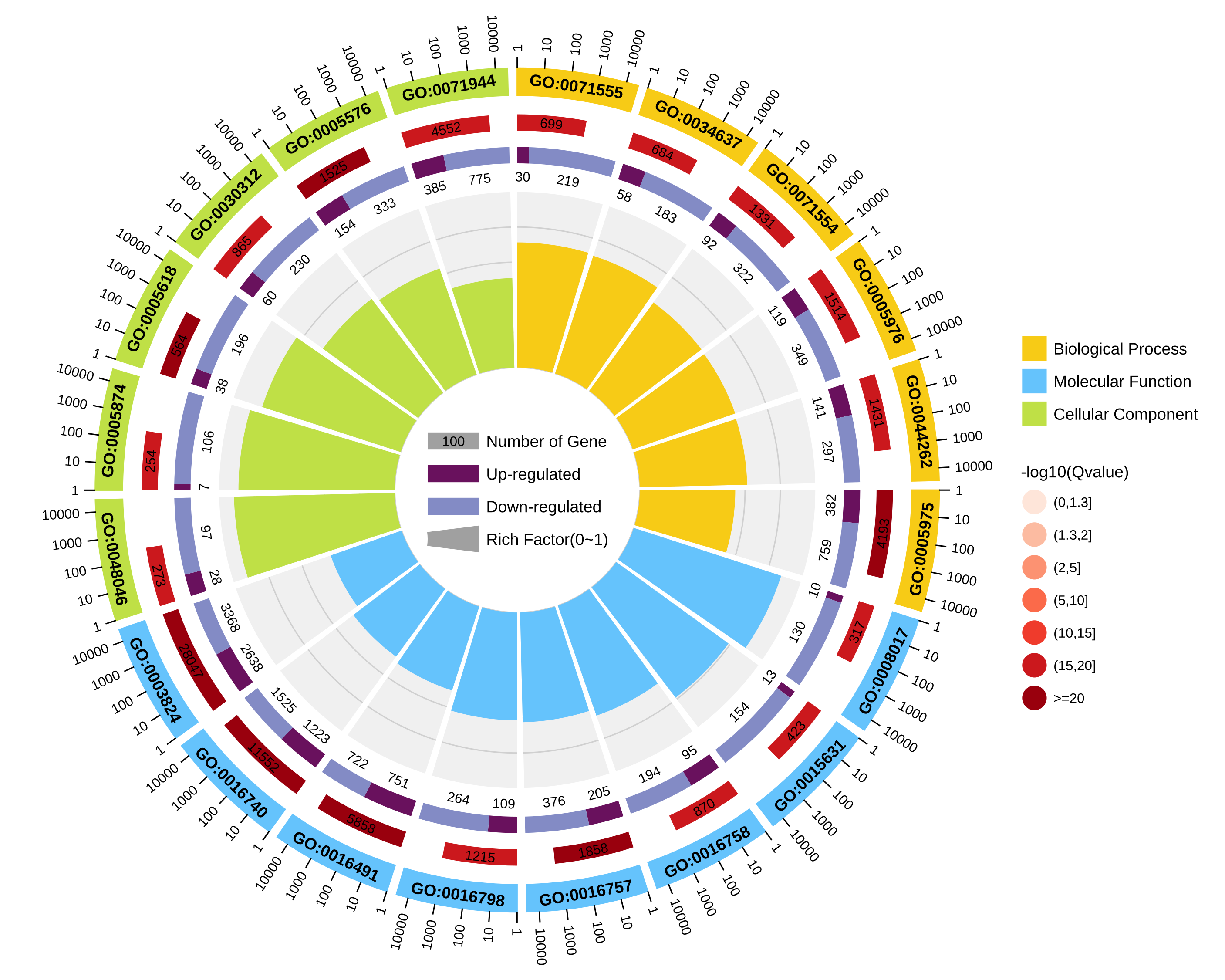
**

**Fig. S5 GO enrichment analysis of up- and down-regulated genes in susceptible tobacco cultivar at 7 days after bacterial wilt infection.** The top GO terms in three categories are listed at *P* ≤ 0.05.


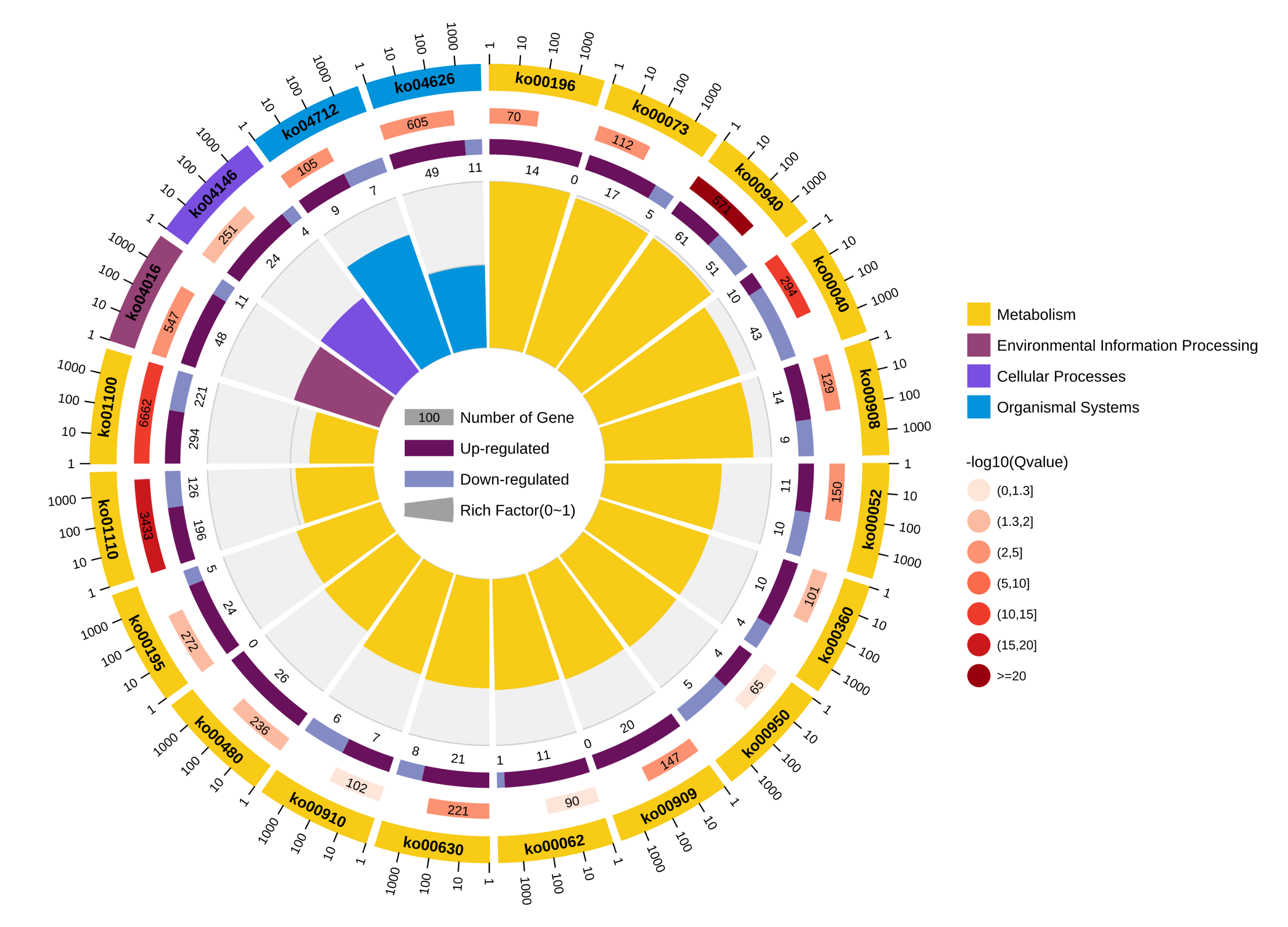


**Fig. S6 KEGG enrichment analysis of up- and down-regulated genes in resistant tobacco cultivar at 3 days after bacterial wilt infection.** The top GO terms in three categories are listed at *P* ≤ 0.05.

**
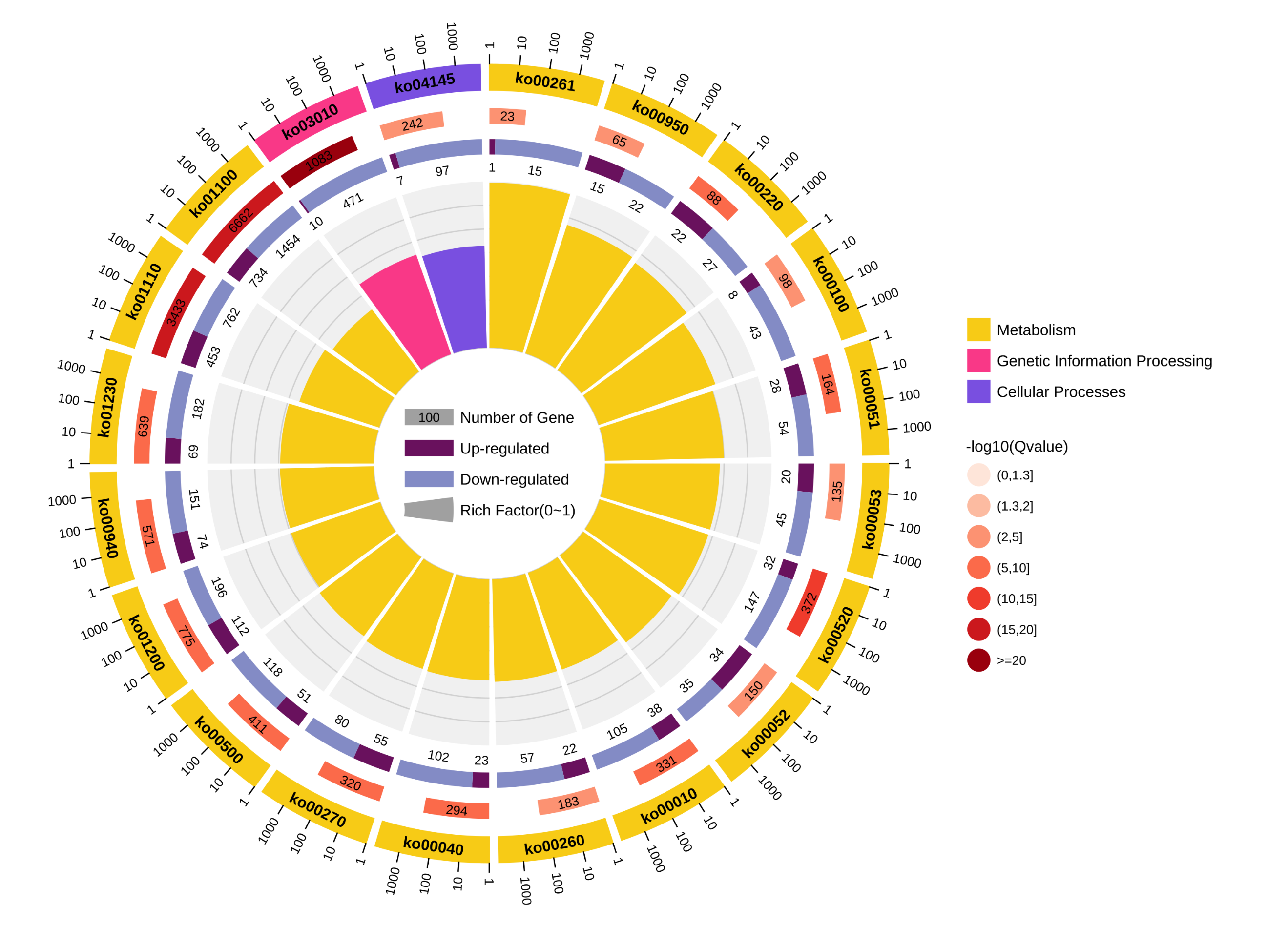
**

**Fig. S7 KEGG enrichment analysis of up- and down-regulated genes in resistant tobacco cultivar at 7 days after bacterial wilt infection.** The top GO terms in three categories are listed at *P* ≤ 0.05.

**
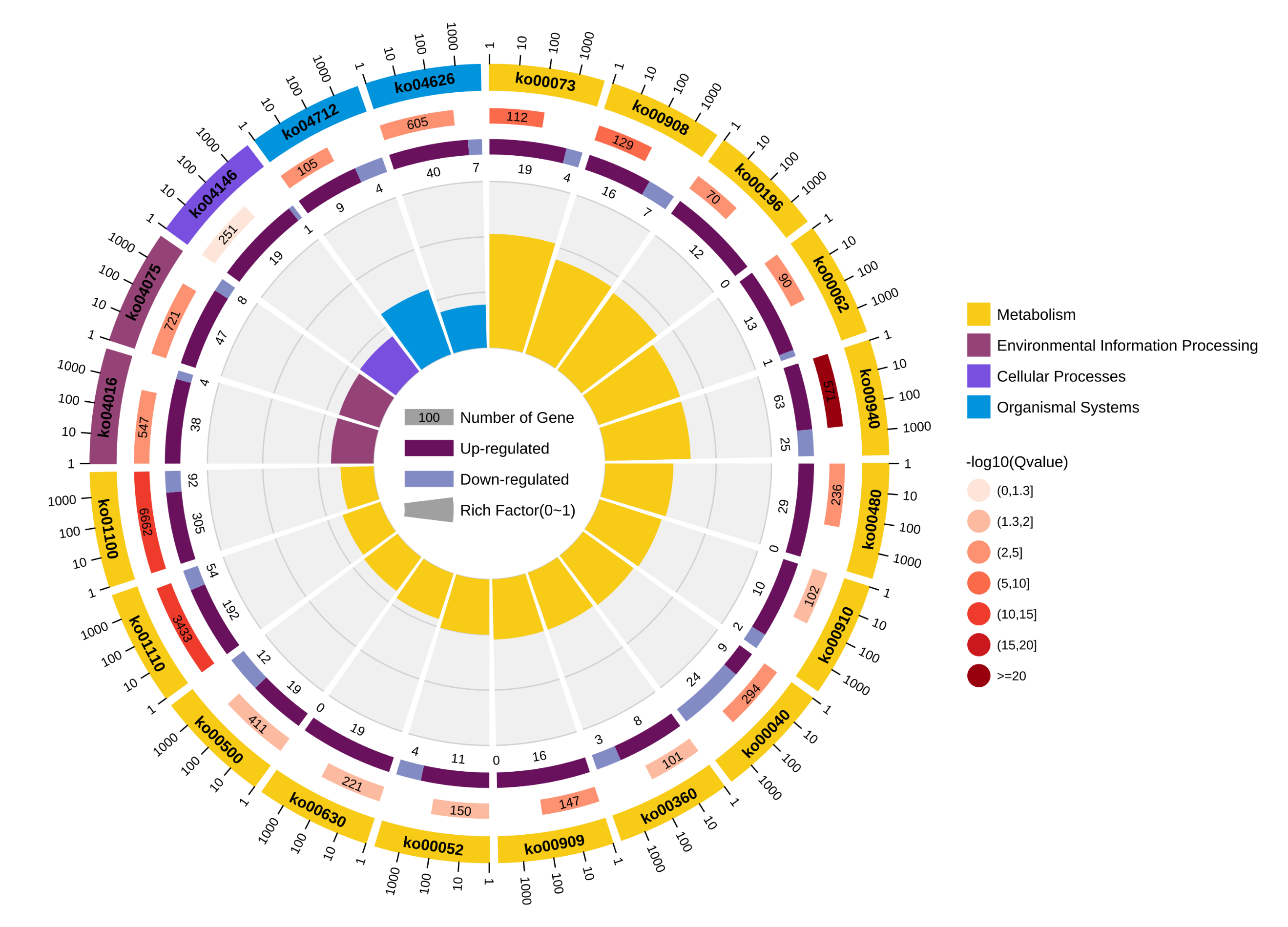
**

**Fig. S8 KEGG enrichment analysis of up- and down-regulated genes in susceptible tobacco cultivar at 3 days after bacterial wilt infection.** The top GO terms in three categories are listed at *P* ≤ 0.05.

**
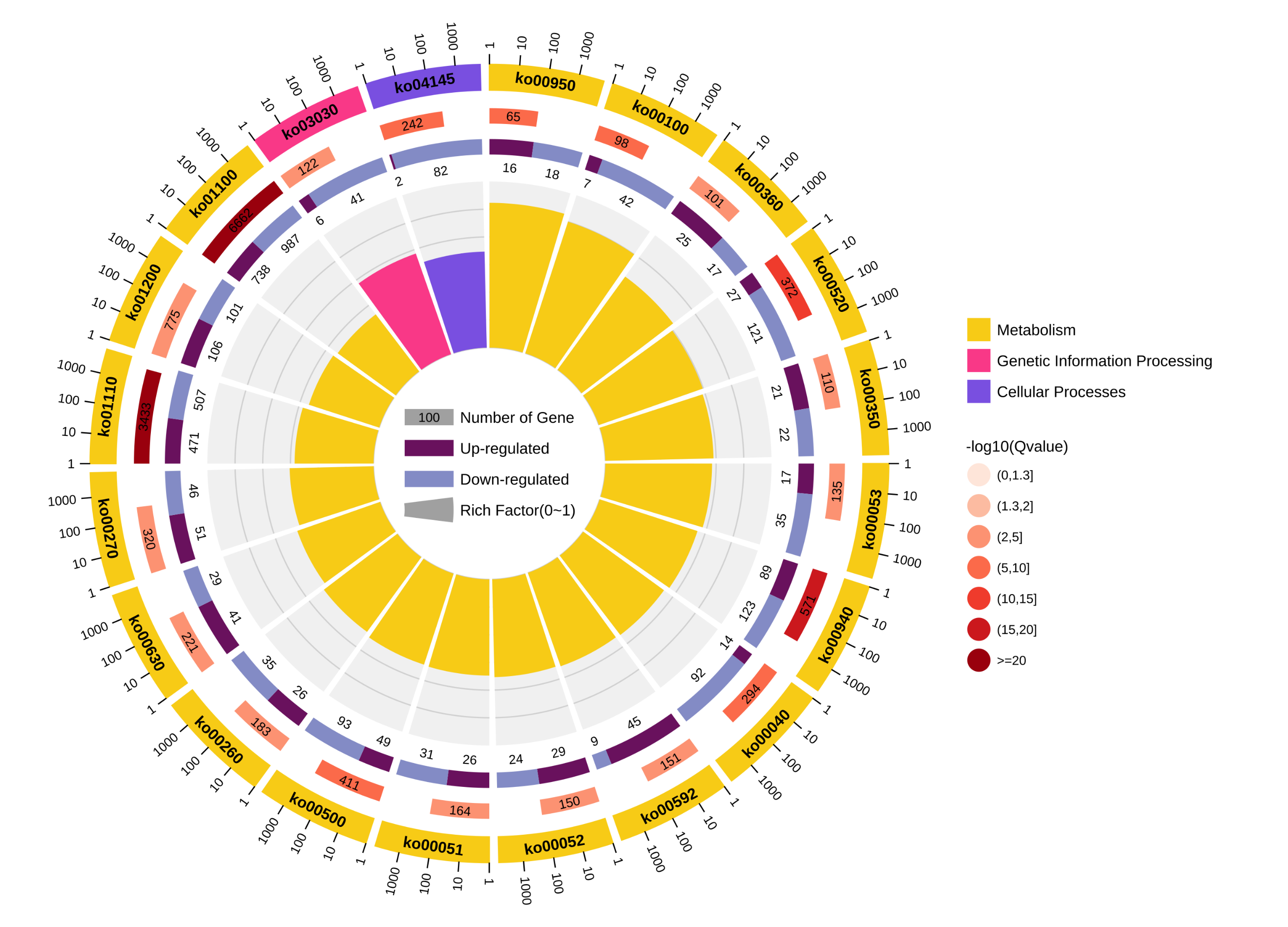
**

**Fig. S9 KEGG enrichment analysis of up- and down-regulated genes in susceptible tobacco cultivar at 7 days after bacterial wilt infection.** The top GO terms in three categories are listed at *P* ≤ 0.05.
